# Three Rings Schiff Base Ester Liquid Crystals: Experimental and Computational Approaches of Mesogenic Core Orientation Effect, Heterocycle Impact

**DOI:** 10.3390/molecules27072304

**Published:** 2022-04-01

**Authors:** Shady Nada, Mohamed Hagar, Omaima Farahat, Ahmed A. Hasanein, Abdul-Hamid Emwas, Abeer Ali Sharfalddin, Mariusz Jaremko, Mohamed A. Zakaria

**Affiliations:** 1 Department of Chemistry, Faculty of Science, Alexandria University, Alexandria 21321, Egypt; shadyadel4869@gmail.com (S.N.); oom_farahat@yahoo.com (O.F.); ahmedhasanein@alexu.edu.eg (A.A.H.); mohamed.zakaria@alexu.edu.eg (M.A.Z.); 2 Core Labs, King Abdullah University of Science and Technology, P.O. Box 4700, Thuwal 23955-6900, Saudi Arabia; abdelhamid.emwas@kaust.edu.sa; 3 Department of Chemistry, Faculty of Science, King Abdulaziz University, P.O. Box 80203, Jeddah 21589, Saudi Arabia; sharfalddin.aa@hotmail.com; 4 Smart-Health Initiative (SHI) and Red Sea Research Center (RSRC), Division of Biological and Environmental Sciences and Engineering (BESE), King Abdullah University of Science and Technology (KAUST), P.O. Box 4700, Thuwal 23955-6900, Saudi Arabia; mariusz.jaremko@kaust.edu.sa

**Keywords:** Schiff base ester liquid crystals, DFT, heterocycles impact

## Abstract

Three rings 2-hydroxypyridine liquid crystalline compounds have been prepared and fully characterized. The mesomorphic behavior of the prepared compounds has been investigated in terms of differential scanning calorimetry (DSC) and polarized optical microscopy (POM). Moreover, a comparative study between the prepared compounds and previously reported analogs has been discussed in terms of the orientation and position of the mesogenic core, in addition to the direction of the terminal alkyl chains. Furthermore, a detailed computational approach has been studied to illustrate the effect of geometrical and dimensional parameters on the type of the enhanced texture and the mesomorphic range and stability. The results of the DFT study revealed that the orientation of the mesogen could affect the mesomorphic behavior and this has been attributed in terms of the degree of the polarizability of the linking groups. This result has been confirmed by calculation of the net dipole moment and the molecular electrostatic potential that show how the mesogen orientation and position could impact the molecular charge separation. Finally, the effect of the pyridyl group has been also investigated in terms of the calculated aromaticity index and the π-π stacking.

## 1. Introduction

Liquid crystals (LCs) represent a state of matter that is thermodynamically placed between the solid phase and liquid phase [1]. Commonly, LC is called a mesomorphic state [2] thus, they have combined properties of them yet expressed special electro-optic phenomena which are unmatched in crystals or liquids. A usual liquid crystal molecule is represented by two main components: a mesogen which is a central rigid part and a spacer which represents the flexible side chains. Furthermore, the rod-like structure consists of a central linkage part between two ring systems [3]. Meanwhile, the physical properties are influenced by the orientation of the anisotropic molecules. Changing the orientation could cause altering in the mechanical properties of the medium and its optical characteristics. This is the main factor that many devices that utilized liquid crystal are based on, such as display panels [4,5]. Moreover, LCs are used recently in optical sensors for imaging trypsin activity [6], organic photovoltaic cells, transistors that benefit from the self-assembly feature [7,8], light polarization [9] and artificial muscles that are employing elastomers, which were formed by weakly cross-linking thermotropic liquid crystal phase with reactive mesogens [10].

Recently, Schiff base LCs widely attracted the attention of the LC community because of the feasibility and low cost of synthetic procedures. They are generally synthesized through the condensation reaction between carbonyl compounds and primary amines to form the (-CH=N-) imine linking group connecting the rigid core components [11]. Additionally, they could offer higher stability and allow mesophase formation [12] which would lead to interesting results such as the formation of room temperature ferroelectric liquid crystals [13], solar energy applications [14], and high efficient flame-retardation and anti-dripping action for polyesters [15]. The ester and imine linking units are the basic structural components for producing the mesomorphism of three aromatic rings thermotropic liquid crystals [16].

In our previous work, we had studied the impact of different lateral substituents [17] and the effect of the proportion of dialkoxy chain length [18] on the mesophase behaviour. We also synthesized new natural fatty acids and observed that mesophases depend on the length of the terminal alkenyl fatty acid chains and were highly impacted by their conformation [19]. Moreover, we had found that Schiff base compounds that contain two rings were mesomorphic except the NO_2_ and the unsubstituted derivatives [20] and we synthesized four rings of Schiff bases/esters and extensively studied their mesophase behavior [21]. It was noticed that the nature of terminal side chains affected the mesomorphic properties of thermotropic calamitic liquid crystals [22].

**Figure 1:**
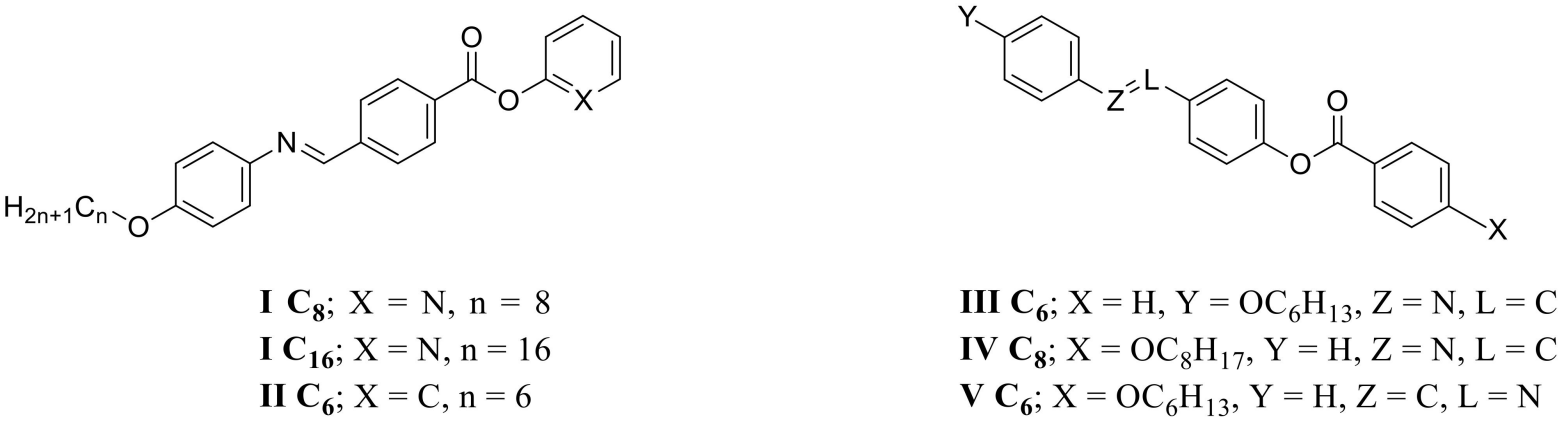
The structures of investigated compounds **I C_8_**, **I C_16_**, **II C_6_**, **III C_6_**, **IV C_8_**, and **V C_6_**.

In order to continue our study on Schiff bases with esters, here in the present work we will investigate the type and orientation of the mesogens and study their effect upon the mesophase stability in addition to the effect of the orientation of terminal groups. The investigation will include a computational DFT study. We will do a comparative study between newly synthesized compounds labelled **I C_8_** and **I C_16_** and compounds from our previous works labelled **II C_6_** [23], **III C_6_** [24], **IV C_8_** [25], and **V C_6_** [26]. Figure 1 illustrates the structures of the investigated compounds.

## 2. Materials and Methods

### 2.1. Materials

N,N’-dicyclohexylcarbodiimide (DCC) (99%), 4-dimethylaminopyridine (DMAP) (98%), ethanol (95%), hydrochloric acid (38%), dichloromethane (99.8%), and 2-hydroxypyridine (98%) were purchased from Sigma Aldrich (Hamburg, Germany). 4-formylbenzonitrile (95%) and 4-octyloxyaniline (98%) were purchased from Aldrich (St. Louis, MO, USA). 4-n-hexadecyloxyaniline (98%) was purchased from Alfa Aesar (Heyvril, MA, USA). All chemicals were used without further purification.

### 2.2. Synthesis

**Scheme 1:**
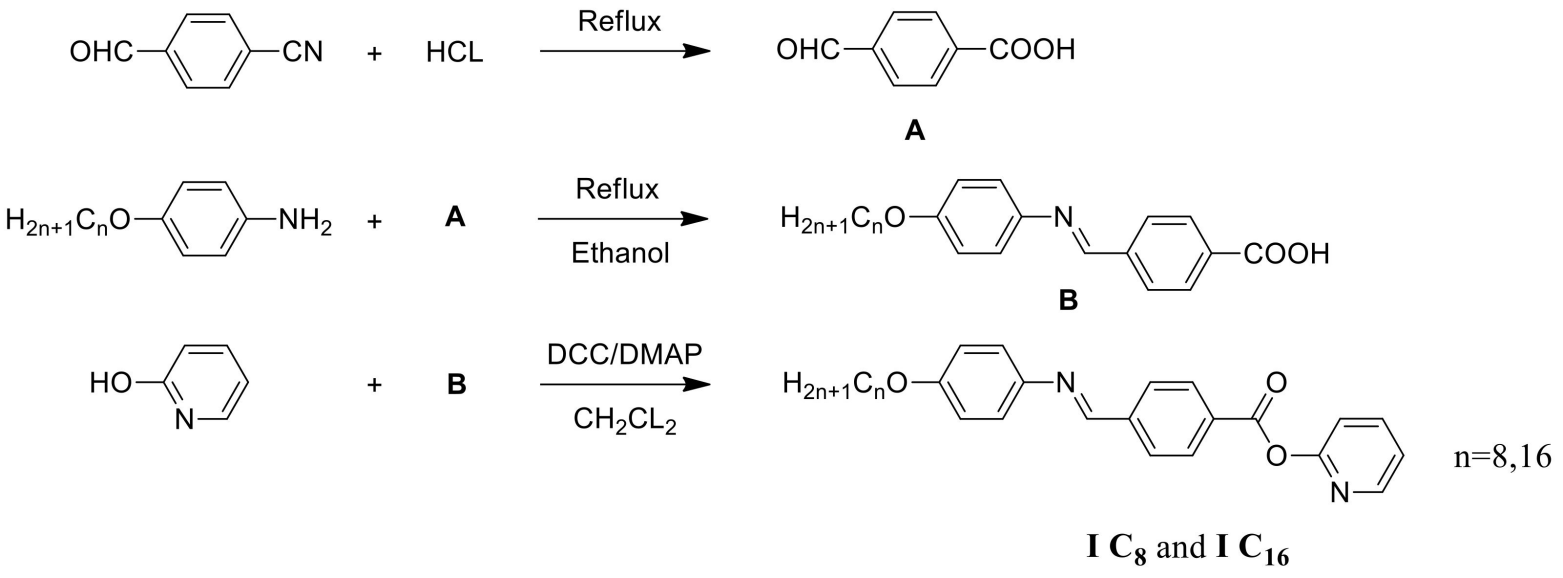
Synthesis of pyridin-2-yl 4-[4-(alkoxy)phenyliminomethyl]benzoate **I C_(8, 16)._**.

The compounds **I C_(8, 16)_** were synthesized according to Scheme 1.

#### 2.2.1. Synthesis of 4-Formylbenzoic Acid

A mixture of 4-formylbenzonitrile (1.0 g, 7.6 mmol) and hydrochloric acid (5 mL) was boiled under reflux for 4 h. The mixture was cooled at room temperature then filtered and washed with cold distilled water. The obtained solid was recrystallized from hot water (see Scheme 1, the top line).

#### 2.2.2. Synthesis of Schiff Base Acid

Equimolar amounts of 4-formylbenzoic acid (500 mg, 3.3 mmol) and 4-alkoxyaniline (3.3 mmol) in ethanol (15 mL) were refluxed for 3 h. The progress of the reaction was monitored using thin-layer chromatography (TLC) on silica gel 60 F_254_ E-Merck (layer thickness 0.2 mm) plates with hexane-ethyl acetate (1:1 *v*/*v*) mobile phase. The spots were visualized under a UV lamp at λ = 254 nm. The resulting mixture was cooled and filtered. The product was washed with ethanol and recrystallized from hot ethanol to give a pure compound (see Scheme 1, the middle line).

#### 2.2.3. Synthesis of Schiff Base Ester

Equimolar amounts of 4-[4-(alkoxy)phenyliminomethyl]benzoic acid (0.3 mmol) and 2-hydroxypyridine (0.3 mmol) were dissolved in dry dichloromethane (10 mL). N,N’-dicyclohexylcarbodiimide (1.2 mmol) and a few 4-dimethylaminopyridine, which was employed as a catalyst, were added to the mixture. The mixture was left under stirring for 72 h at room temperature then filtered to remove the by-product 1,3-dicyclohexyl urea (DCU). The filtrate was left until it completely evaporated then the product was recrystallized from hot ethanol to give a pure compound. The progress of the reaction was monitored using thin-layer chromatography (TLC) on silica gel 60 F_254_ E-Merck (layer thickness 0.2 mm) plates with hexane-ethyl acetate (1:1 *v*/*v*) mobile phase. The spots were visualized under a UV lamp at λ = 254 nm (see Scheme 1, the bottom line).

***Pyridin-2-yl 4-[4-(octyloxy)phenyliminomethyl]benzoate (I C_8_):***

Yield: 81.0%, FTIR (ύ, cm^−1^): 2947–2854 (CH_2_ stretching), 1728 (C=O), 1605 (C=N), 1589 (C=C), 1466 (C-O_Asym_), 1250 (C-O_Sym_). ^1^H NMR (400 MHz, CDCl_3_): δ/ppm: 0.92 (t, 3 H, *J* = 8 Hz, CH_3_(CH_2_)_4_CH_2_CH_2_CH_2_), 1.31–1.38 (m, 8 H, CH_3_(CH_2_)_4_CH_2_CH_2_CH_2_), 1.46–1.49 (m, 2 H, CH_3_(CH_2_)_4_CH_2_CH_2_CH_2_), 1.79–1.84 (m, 2 H, CH_3_(CH_2_)_4_CH_2_CH_2_CH_2_), 4.00 (t, 2 H, *J* = 8 Hz, CH_3_(CH_2_)_4_CH_2_CH_2_CH_2_), 6.97 (d, 2 H, *J* = 8 Hz, Ar-H), 7.27 (d, 2 H, *J* = 8 Hz, Ar-H), 7.38 (d, 2 H, *J* = 8 Hz, Ar-H), 7.51 (dd, 1 H, *J_1_* = 8, *J_2_* = 4 Hz, Py-H), 8.01 (d, 2 H, *J* = 8 Hz, Ar-H), 8.48–8.54 (m, 2 H, Py-H), 8.90 (dd, 1 H, *J_1_* = 8, *J_2_* = 4 Hz, Py-H), 9.44 (s, 1 H, CH=N), (Supplementary Materials).

***Pyridin-2-yl 4-[4-(hexadecyloxy)phenyliminomethyl]benzoate* (I C_16_):**

Yield: 83.0%, **FTIR (ύ, cm^−1^):** 2916–2854 (CH_2_ stretching), 1728 (C=O), 1605 (C=N), 1589 (C=C), 1466 (C-O_Asym_), 1250 (C-O_Sym_). ^1^H NMR (400 MHz, CDCl_3_): δ/ppm: 0.92 (t, 3 H, *J* = 8 Hz, CH_3_(CH_2_)_12_CH_2_CH_2_CH_2_), 1.32–1.37 (m, 24 H, CH_3_(CH_2_)_12_CH_2_CH_2_CH_2_), 1.48–1.62 (m, 2 H, CH_3_(CH_2_)_12_CH_2_CH_2_CH_2_), 1.81–1.84 (m, 2 H, CH_3_(CH_2_)_12_CH_2_CH_2_CH_2_), 4.00 (t, 2 H, *J* = 8 Hz, CH_3_(CH_2_)_12_CH_2_CH_2_CH_2_), 6.97 (d, 2 H, *J* = 8 Hz, Ar-H), 7.28 (d, 2 H, *J* = 8 Hz, Ar-H), 7.37 (d, 2 H, *J* = 8 Hz, Ar-H), 7.52 (dd, 1 H, *J_1_* = 8, *J_2_* = 4 Hz, Py-H), 8.01 (d, 2 H, *J* = 8 Hz, Ar-H), 8.48–8.53 (m, 2 H, Py-H), 8.90 (dd, 1 H, *J_1_* = 8, *J_2_* = 4 Hz, Py-H), 9.44 (s, 1 H, CH=N) (Supplementary Materials).

The phase changes in the materials were determined via differential scanning calorimetry (DSC), DSC-60A, Shimadzu, Japan. Specimens of the size 2–3 mg were encapsulated in aluminium pans and were heated or cooled under a dry nitrogen atmosphere. Measurements were performed at 10.0 °C/min. Samples were heated from room temperature to 200 °C and cooled back to room temperature at the same heating rate, all under an inert nitrogen gas atmosphere. The phase transition temperature values were determined from the endothermic peak minima of enthalpy in the heating curves. The accuracy of temperature monitoring was better than 1.0 °C.

Transition temperatures for the prepared compounds were checked and phases were identified by polarized optical microscope (POM, Wild, Germany) attached with Mettler FP82HT hot stage.

## 3. Results and Discussion

### 3.1. Mesomorphic Behaviour

**Figure 2:**
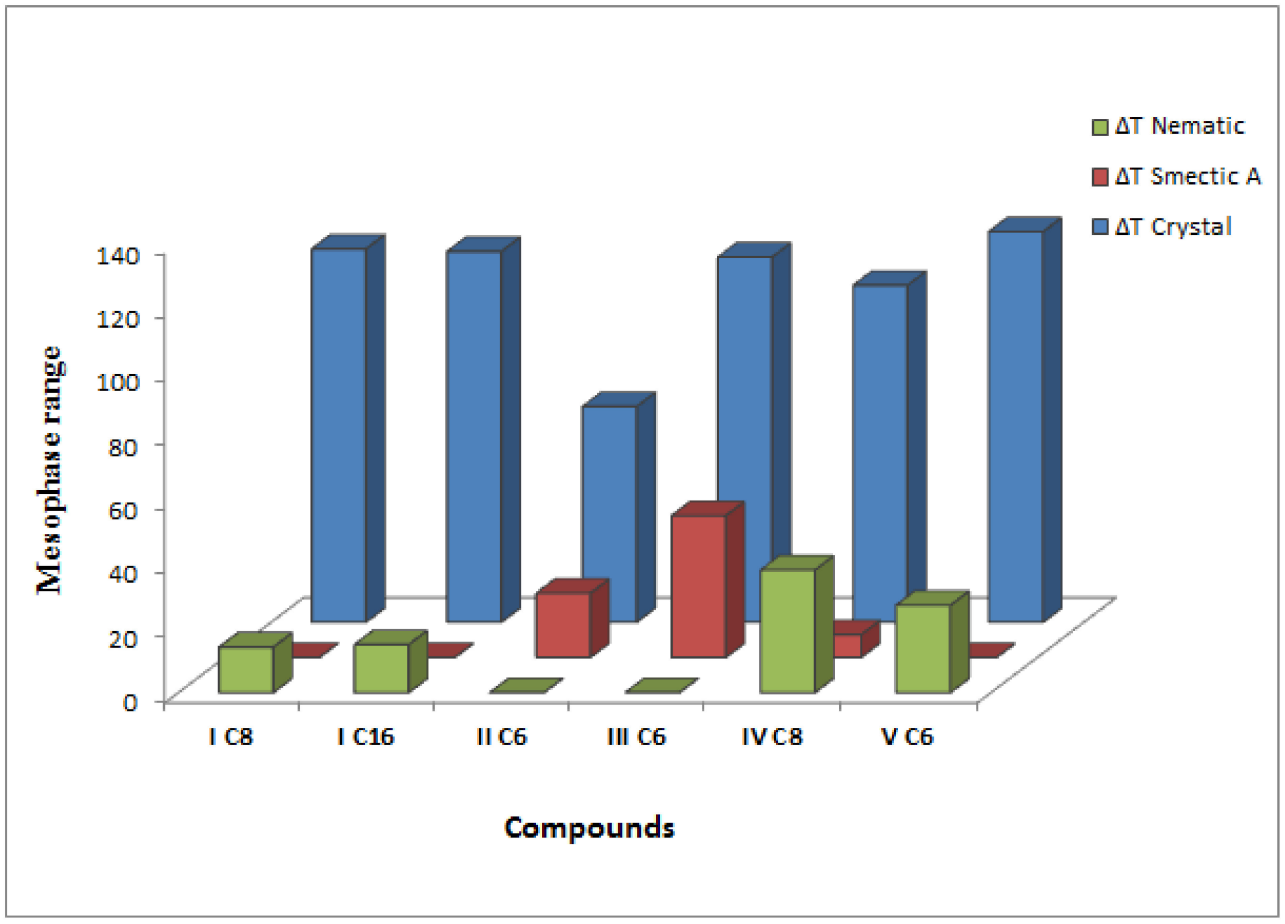
Mesophase ranges of compounds **I C_8_**, **I C_16_**, **II C_6_**, **III C_6_**, **IV C_8_**, and **V C_6_**.

**Figure 3:**
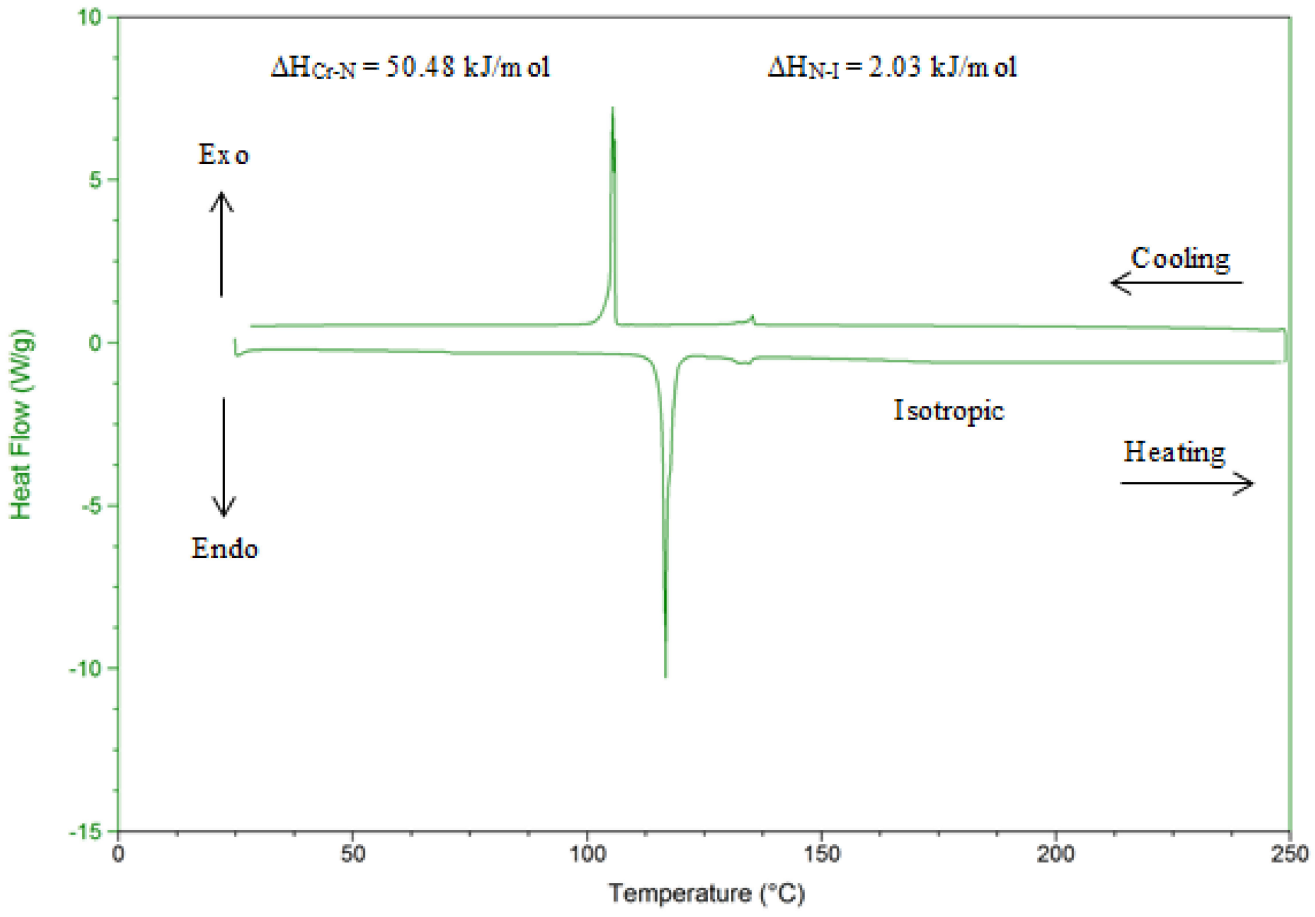
Differential scanning calorimetry (DSC) thermogram of compounds **I C_8_** during heating and cooling cycles, at 10.0 °C/min.

**Figure 4:**
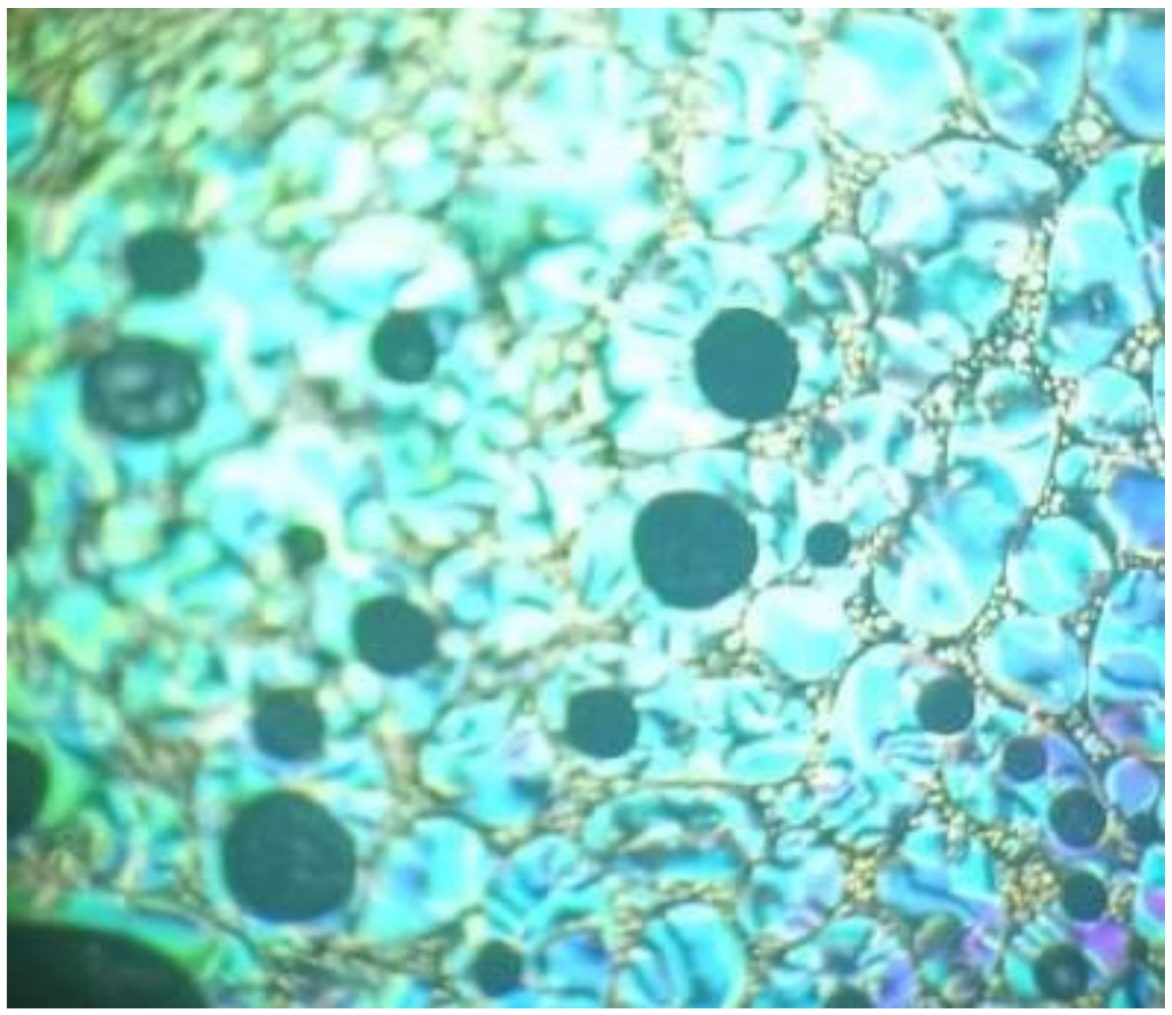
Nematic texture under polarized optical microscopy (POM) upon heating for **I C_16_** at 122 °C, at 10.0 °C/min.

The investigated compounds were characterized for their mesomorphic behavior by differential scanning calorimetry (DSC) and polarized optical microscopy (POM). The types of mesophases were identified by POM. The phase transition temperatures (°C), enthalpy of transition ΔH (kJ/mol), and normalized entropy of transition ΔS/R were measured by DSC. All the results are tabulated in Table 1 and the mesophase ranges are graphically represented in Figure 2. An indicative example demonstrating the DSC curve of the heating/cooling cycles for **I C_8_** is shown in Figure 3. The presence of sharp peaks in the DSC thermogram indicates the phase changing between the crystal and the liquid crystalline. Additionally, the small peaks represent the transition from the liquid crystalline phase to the isotropic liquid. The type of mesophase texture for **I C_16_** is represented in Figure 4.

Table 1 showed the phase transition temperatures (°C) at 10.0 °C/min, enthalpy of transition ΔH (kJ/mol), the entropy of transition ΔS (J/mol.K), and the normalized entropy of transition of compounds **I C_8_**, **I C_16_**, **II C_6_**, **III C_6_**, **IV C_8_**, and **V C_6_**. It was obvious that all the compounds have a wide range of mesophase stability, thus, they are mesogenic in nature. **IV C_8_** is the only dimorphic compound that exhibited in smectic A and nematic mesophases with mesomorphic ranges of 7.1°C and 38.2 °C, respectively. The smectic A-ranges decreased in order **III C_6_** (44.2 °C) > **II C_6_** (20 °C) > **IV C_8_** (7.1 °C). Additionally, the nematic ranges increased in order **I C_8_** (14.17 °C) < **I C_16_** (14.96 °C) < **V C_6_** (27.2 °C) < **IV C_8_** (38.2 °C). These trends were affected by the position and orientation of the mesogenic core. On the other hand, **V C_6_** had the highest melting temperature (Cr-N) with 121.8 °C, and **II C_6_** (Cr-SmA) had the lowest one with 67.3 °C. The development of SmA mesophase in **IV C_8_** and not in **V C_6_** was probably due to changes in the orientation of the mesogenic (-CH=N-) as it increased the dipole moment for **IV C_8_** over **V C_6_**. The results of the dipole moment will be discussed later as one of the important factors affecting mesomorphic behavior. On the same approach, the existence of SmA mesophase in **II C_6_** and not in **I C_8_** nor **I C_16_** could be explained in terms of the dipole moment where the higher dipole moment could enhance the smectic mesophase [27,28].

**Table 1:** Phase transition temperatures (°C) at 10.0 °C/min., enthalpy of transition ΔH (kJ/mol), the entropy of transition ΔS (J/mol.K), and normalized entropy of transition of compounds **I C_8_**, **I C_16_**, **II C_6_**, **III C_6_**, **IV C_8_**, and **V C_6_**, all values have been calculated for the heating transition.

Compounds	T_Cr-SmA_	T_Cr-N_	T_SmA-N_	T_SmA-I_	T_N-I_	ΔH_Cr-N_	ΔS_Cr-N_	ΔS_Cr-N_/R	ΔH_Cr-SmA_	ΔS_Cr-SmA_	ΔS_Cr-SmA_/R	ΔH_N-I_	ΔS_N-I_	ΔS_N-I_/R	ΔH_SmA-I_	ΔS_SmA-I_	ΔS_SmA-I_/R
**I C_8_**	-	116.42	-	-	130.59	50.48	129.58	15.58	-	-	-	2.03	5.03	0.60	-	-	-
**I C_16_**	-	115.65	-	-	130.61	69.48	178.70	21.49	-	-	-	4.51	11.17	1.34	-	-	-
**II C_6_** [23]	67.3	-	-	87.3	-	-	-	-	28.38	83.36	10.02	-	-	-	1.92	5.33	2.65
**III C_6_** [24]	113.9	-	-	158.1	-	-	-	-	36.39	94.01	11.30	-	-	-	0.95	2.20	0.26
**IV C_8_** [25]	105.1	-	112.2	-	150.4	-	-	-	-	-	-	0.3	0.7	0.08	-	-	-
**V C_6_** [26]	-	121.8	-	-	149.0	-	-	-	-	-	-	0.81	1.91	0.23	-	-	-

Cr-SmA = Crystal to smectic A transition; Cr-N = Crystal to nematic transition; SmA-I = Smectic A to isotropic liquid; SmA-N = Smectic A to nematic transition; N-I = Nematic to isotropic liquid transition.

### 3.2. DFT Calculations

#### 3.2.1. The Geometrical Structure

**Figure 5:**
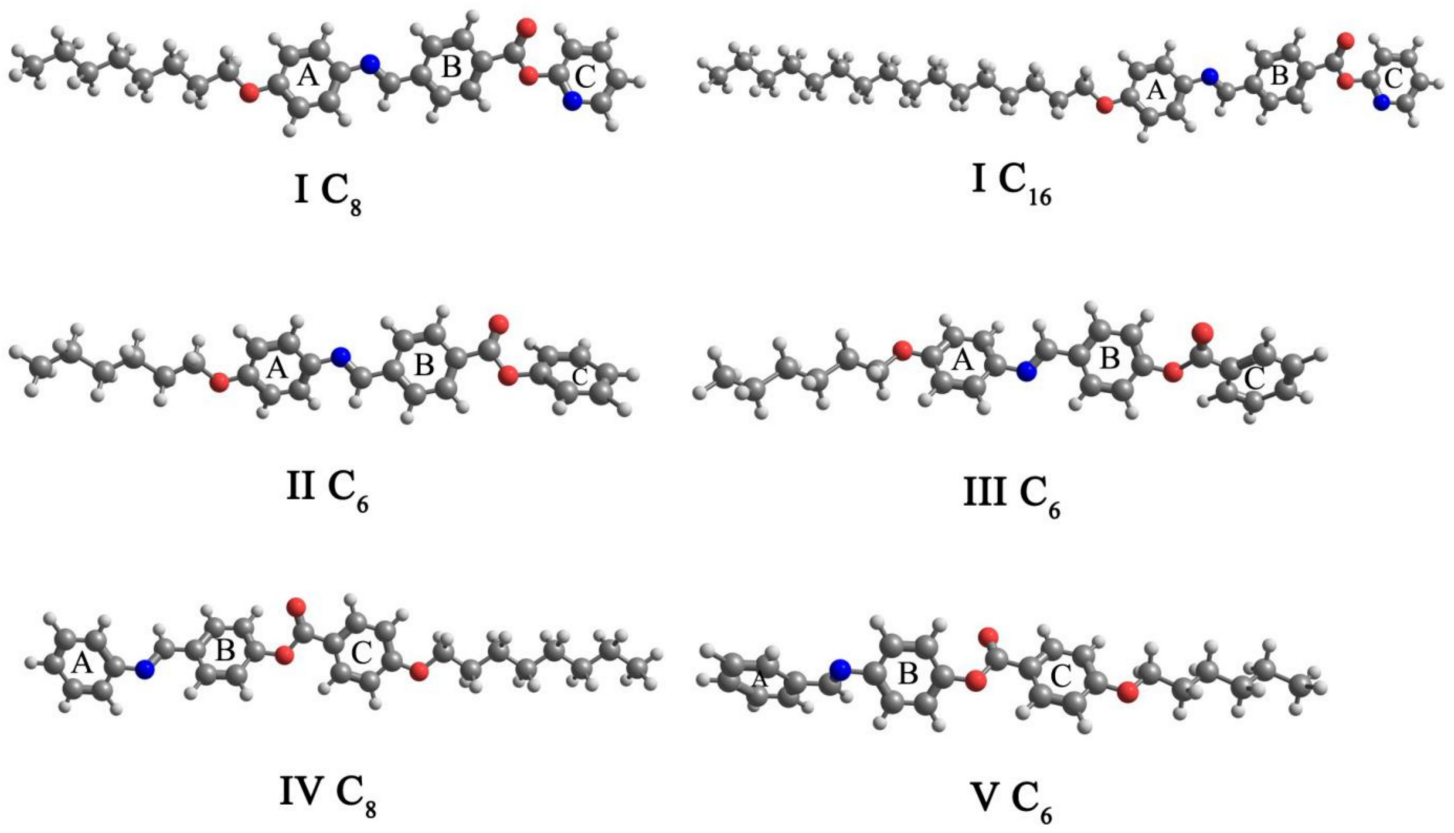
Calculated molecular geometrical structures of compounds **I C_8_**, **I C_16_**, **II C_6_**, **III C_6_**, **IV C_8_**, and **V C_6_** (blue—N, red—O, white—H, grey—C).

**Figure 6:**
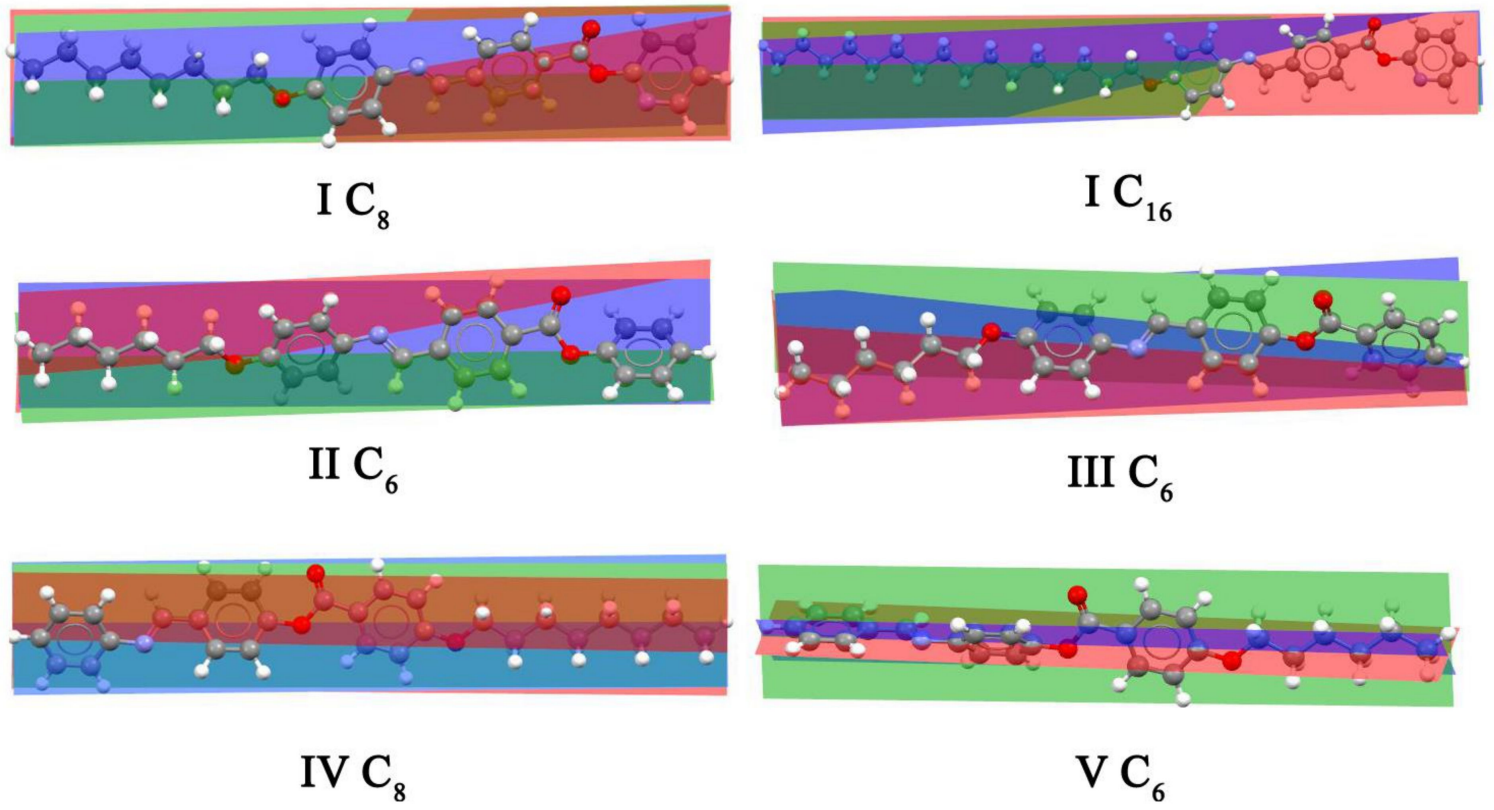
Estimated geometrical structure and twist angles of compounds **I C_8_**, **I C_16_**, **II C_6_**, **III C_6_**, **IV C_8_**, and **V C_6_**.

The optimum geometrical structure of all compounds was calculated using Gaussian 09 software [29] and performed using the DFT/B3LYP method using a 6–311G(d,p) basis set. The geometry for each compound was optimized to find the geometrical structure for the minimum energy of conformations regarding all geometrical parameters, then, the optimized structures were used in the estimation of the frequency. The optimized molecular geometrical structures are illustrated in Figure 5. The twist angles of compounds are tabulated in Table 2 and illustrated in Figure 6, where the red, blue and green planes are passing through the rings A, B and C, respectively. It was observed from the results of the DFT calculations of all compounds that the three rings A, B, and C were non-co-planer. The deviation from planarity could be related to the position of the mesogenic cores (-CH=N- and COO) of the liquid crystalline compounds. It is observed from Table 2 that twist angles between A and C rings in **I C_8_** (6.63°) and **I C_16_** (9.04°) compounds were much lower than their analogous phenyl compound **II C_6_** (79.97°). One could conclude from the previous observation that the presence of nitrogen atoms enhanced the planarity which increased the mesophase temperature.

The different lengths and widths of the investigated compounds resulted in the aspect ratios and areas which are tabulated in Table 3. Aspect ratio is the ratio of width to height while the area represents the amount or extent of surface which equals to the multiply of the width by the height. These quantities demonstrated the collision diameter of the compounds. The compounds with higher aspect ratios were more likely to have stronger terminal and lateral interactions as the space-filling of the liquid crystals compounds increased. Compound **C_16_**, which had the longest alkoxy chain, possessed the highest aspect ratio value. The order of increasing aspect ratios was **III C_6_** < **II C_6_** < **V C_6_** < **I C_8_** < **IV C_8_** < **I C_16_**.

It was noticed that changing the orientation of the mesogenic core (-CH=N-) between **IV C_8_** and **V C_6_** may lead to a very small change in the aspect ratio. However, changing the position of the alkoxy terminal chain altered the aspect ratio significantly as seen in **III C_6_** and **V C_6_**.

**Figure 7:**
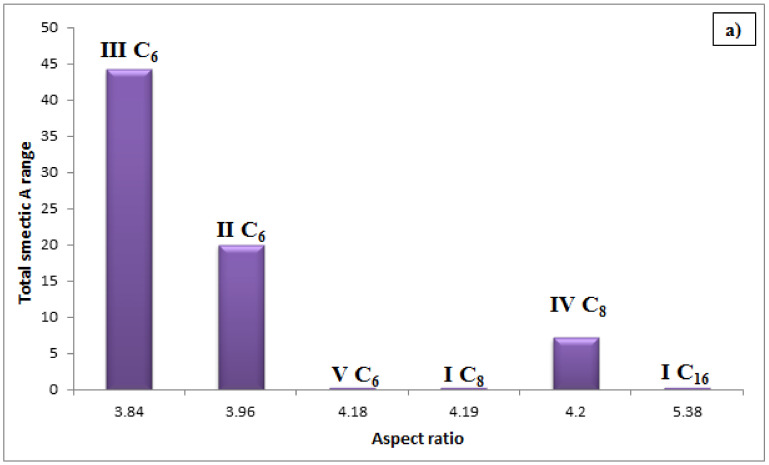

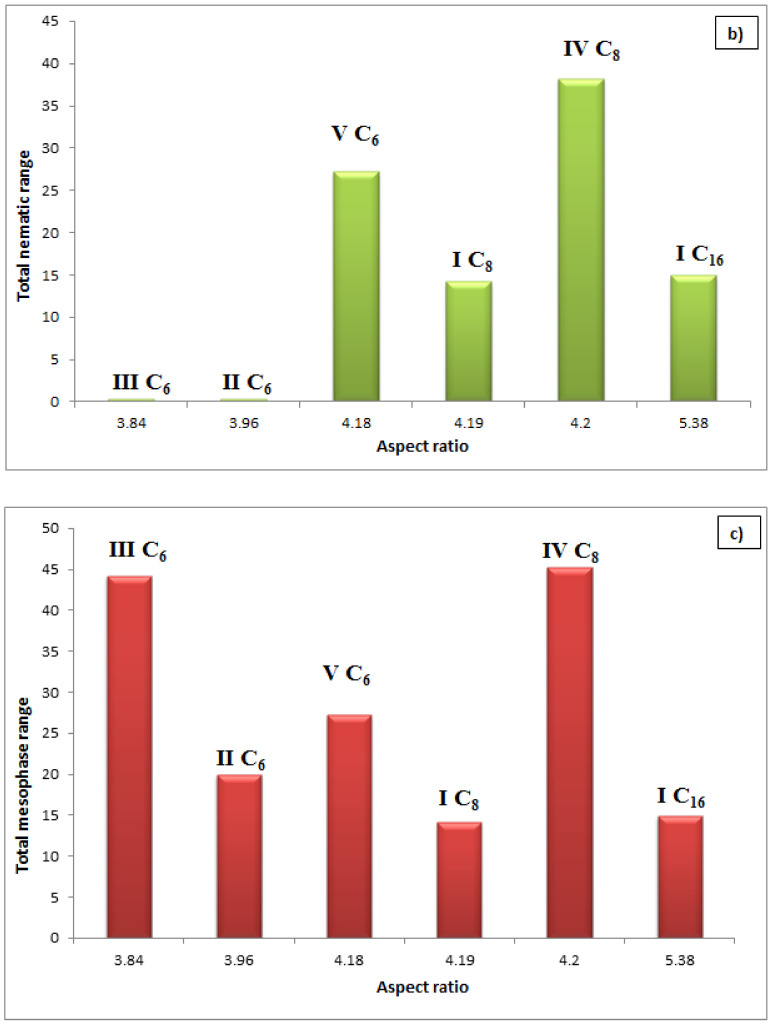
Dependence of aspect ratio with (**a**) Smectic range (**b**) Nematic range (**c**) Total mesophase range of compounds **I C_8_**, **I C_16_**, **II C_6_**, **III C_6_**, **IV C_8_**, and **V C_6_**.

It is noticed from Figure 7 that only compounds **II C_6_**, **III C_6,_** and **IV C_8_** have a smectic A mesophase. It is worth mentioning that changing the orientation of the mesogenic core (COO) in **II C_6_** and **III C_6_** may alter the aspect ratio value. Compound **II C_6_** had a higher aspect ratio than compound **III C_6_** with the same chain length (C = 6), but the smectic A mesophase range was lower in compound **II C_6_** (ΔT = 20 °C) compared to what **III C_6_** had (ΔT = 44.2 °C).

#### 3.2.2. Molecular Electrostatic Potentials (MEP)

**Figure 8:**
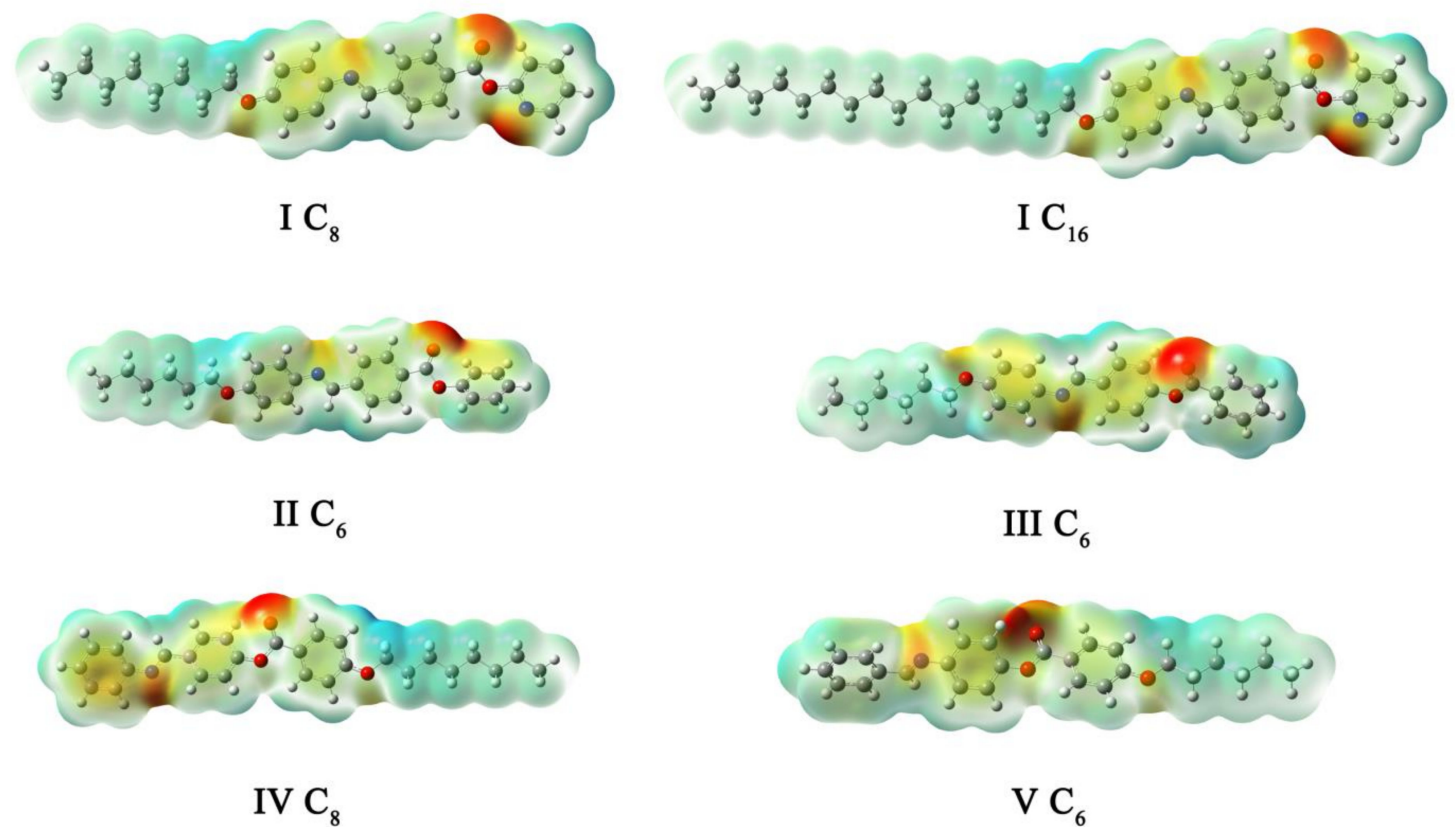
The estimated molecular electrostatic potentials (MEP) of compounds **I C_8_**, **I C_16_**, **II C_6_**, **III C_6_**, **IV C_8_**, and **V C_6_**.

Charge distribution maps were estimated according to the molecular electrostatic potential (MEP) [30] for compounds **I C_8_**, **I C_16_**, **II C_6_**, **III C_6_**, **IV C_8_**, and **V C_6_** which were calculated by the DFT/B3LYP method using a 6–311G(d,p) basis set and provided in Figure 8. The electron density increased in the order red > orange > yellow > green > blue, therefore, the most negatively charged sites, which are illustrated in red color, were the high electronegative oxygen atoms and nitrogen atoms. The maximum was the carbonyl oxygen atom as well as the nitrogen atom in the pyridine ring (for **I C_8_** and **I C_16_**) which will be more likely to react with the electrophilic reagent. The minimum negatively charged sites are illustrated in a blue color, where the first carbon of the terminal alkoxy chains is more probable to be attacked with nucleophiles. Expectedly, it was noticed that the length of the alkyl chain (in **I C_8_** and **I C_16_**) had an insignificant effect on the distribution of the electron density on the remaining part of the compound. The orientation of the charge distribution could have an impact on the degree of packing of the compounds which will affect their mesophase. It was observed that the negative charge was localized in the center of compounds **III C_6_**, **IV C_8_**, and **V C_6_** which was expected to permit a high degree of packing. Additionally, the high degree of charge separation in **III C_6_** and **IV C_8_** compounds predicted high molecular packing which influenced the mesophase ranges.

#### 3.2.3. Frontier Molecular Orbitals (FMOs)

**Figure 9:**
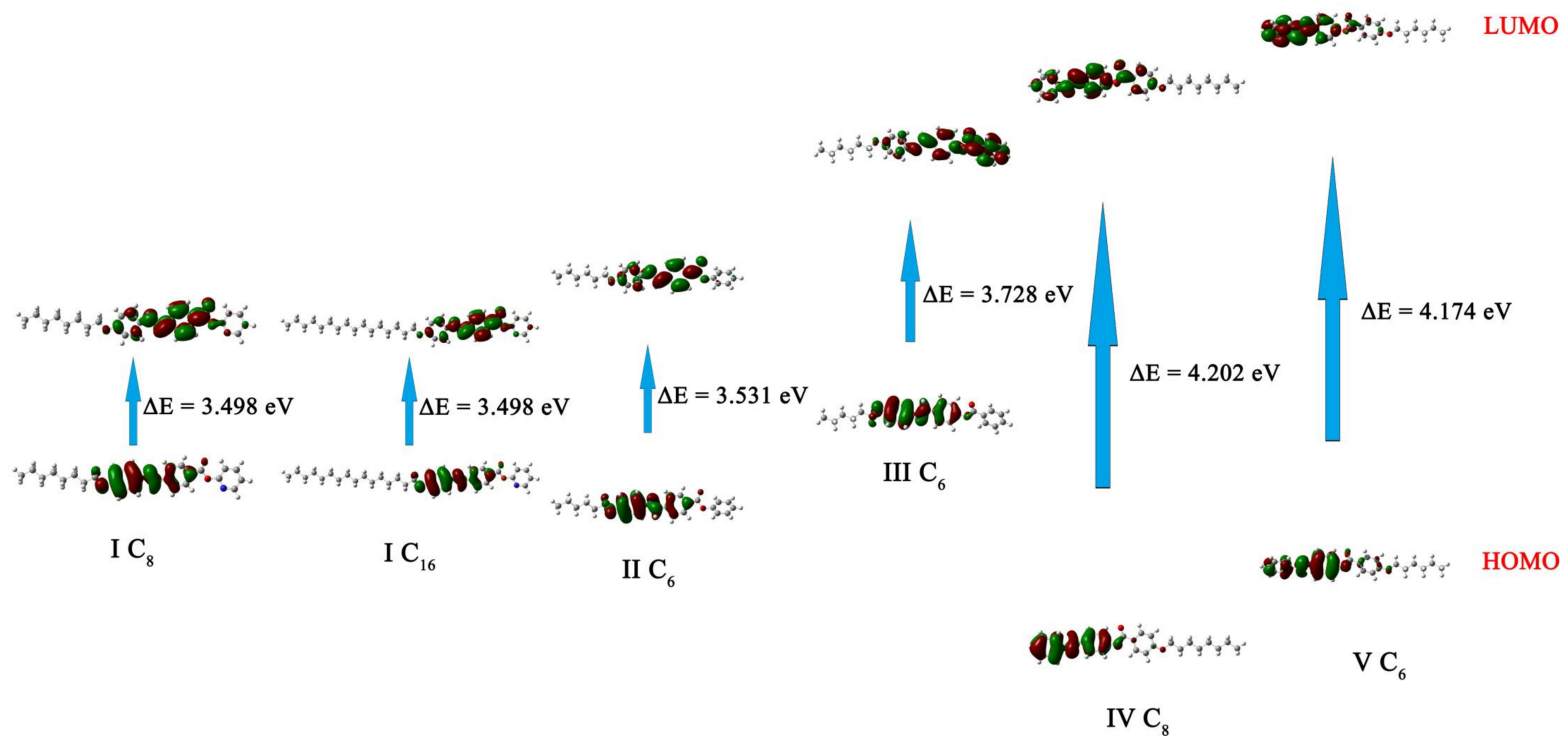
The estimated frontier molecular orbitals of compounds **I C_8_**, **I C_16_**, **II C_6_**, **III C_6_**, **IV C_8_**, and **V C_6_**.

Frontier molecular orbitals (FMOs) are referred to as the highest occupied molecular orbital (HOMO) and the lowest unoccupied molecular orbital (LUMO) [31]. The HOMO is an electron donor while LUMO is an electron acceptor. The frontier molecular orbitals (FMO) can predict the ability of electron transport and determine the reactivity of the molecules. The energies of HOMO and LUMO orbitals of the compounds were calculated with the same method at the same basis set and the results are tabulated in Table 4 and shown in Figure 9.

**Figure 10:**
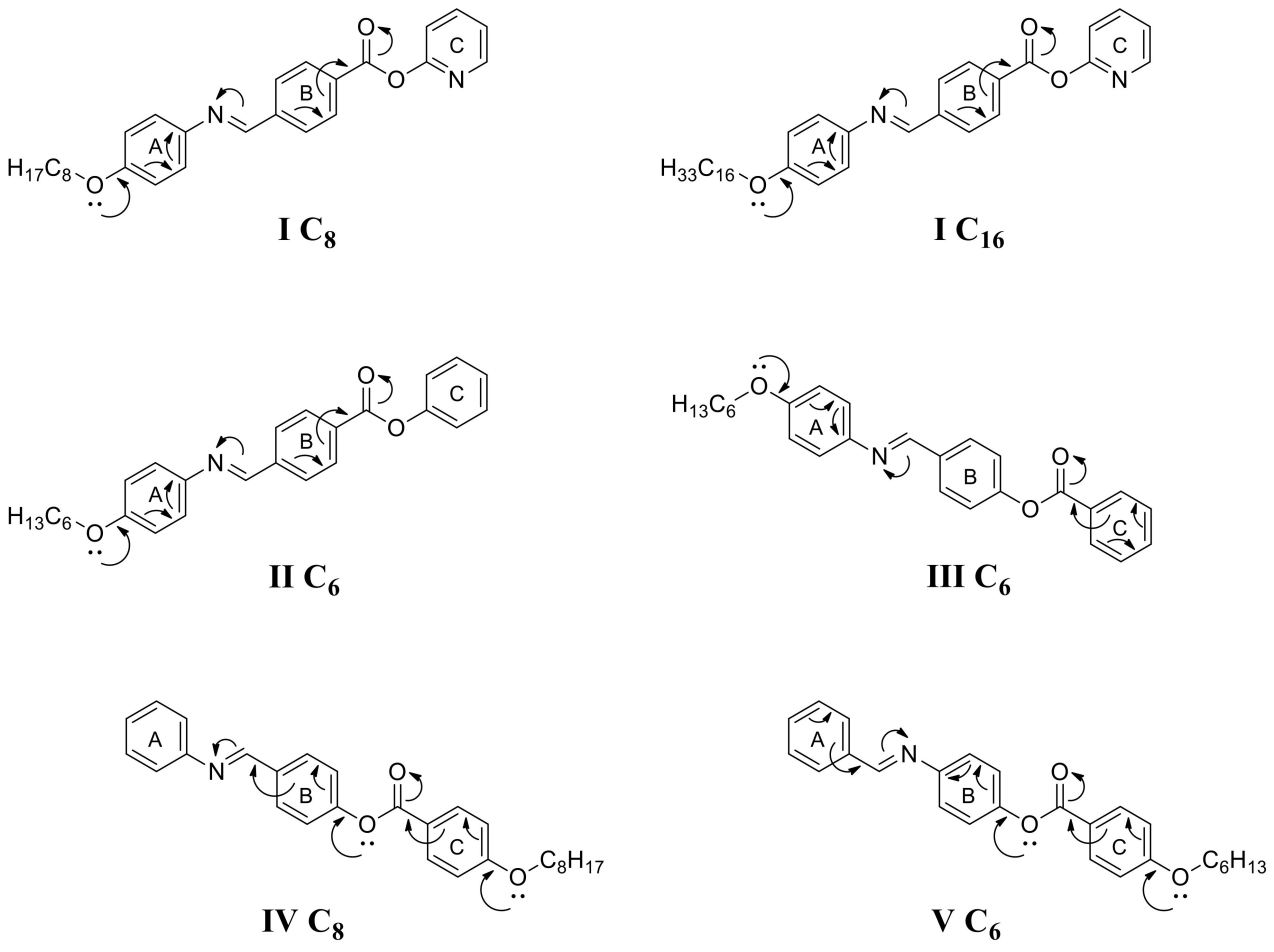
The proposed resonance of compounds **I C_8_**, **I C_16_**, **II C_6_**, **III C_6_**, **IV C_8_**, and **V C_6_**.

It is noticed that the length of the alkoxy chains in **I C_8_** and **I C_16_** had an insignificant effect on the energy gap between the FMOs. The electron densities of the regions that form the HOMO and the LUMO were mostly localized on the aromatic rings. **II C_6_** of the phenyl derivative had a higher energy difference. Since the presence of the N-atom could be the factor affecting the degree of conjugation, introducing nitrogen atoms in **I C_8_** and **I C_16_** lowers the LUMO level and consequently decreased the energy gap which enhanced the electronic transition. Furthermore, it was observed that the energy gap was affected by the position of the mesogenic core (COO) as can be seen in **II C_6_** and **III C_6_**. The position of the carbonyl in **II C_6_** may allow the maximum delocalization of the π-electrons which decreased the energy gap of the FMO. Moreover, the energy gap was higher in **IV C_8_** than **III C_6_** which could be attributed to the presence of higher resonance in **III C_6,_** unlike **IV C_8_** which may face the difficulty of resonance between the high electronegativity nitrogen atom of (-CH=N-) group and the attached aromatic ring. Additionally, there was a slight decrease in the energy gap in **V C_6_** compared with **IV C_8_** which may be a result of the ease of resonance between the carbon atom of the (-CH=N-) group and the aromatic ring as illustrated in Figure 10.

The results of the energy of compounds (Table 4) showed that **I C_16_** was the highest stable compound. The stability of the compounds under study increased in the order **II C_6_** < **III C_6_** < **V C_6_** < **IV C_8_** < **I C_8_** < **I C_16_**.

#### 3.2.4. Dipole Moment and Polarizability

The dipole moments and polarizability were calculated with the same method at the same basis set and tabulated in Table 5. The dipole moment was greatly affected by the orientation and position of the mesogenic core as well as the distance between atoms. It was noticed that **I C_16_** had a slightly higher dipole moment than **I C_8_** which could be attributed to the longer alkyl chains of the terminal alkoxy chains. The dipole moment was increased when the same point charges were separated along a larger distance. Furthermore, it was observed that **II C_6_** had a higher dipole moment than the two earlier mentioned compounds which could be related to the absence of the high electronegativity nitrogen atom of the pyridine ring thus a higher difference in electronegativity along the compound. Meanwhile, the dipole moment was dramatically decreased in **III C_6_** due to the changing position of the carbonyl oxygen which could be related to the occurrence of resonance between the aromatic ring and the carbonyl oxygen that lowers the separation of charges along the compound. **IV C_8_** had the highest dipole moment which could be related to the unidirectional electron delocalization by resonance along the molecular backbone. **V C_6_** had a lower dipole moment due to opposing directions of resonance between central p-phenylene [ring B] with oxygen and nitrogen atoms, resulting in a cancelling effect [32,33].

It is well known that the increment in the dipole moment highly affected the mesophase range [34,35]. It could be explained in terms of the degree of packing of the molecules in the liquid crystalline phases which affect the mesophase stability.

**Figure 11:**
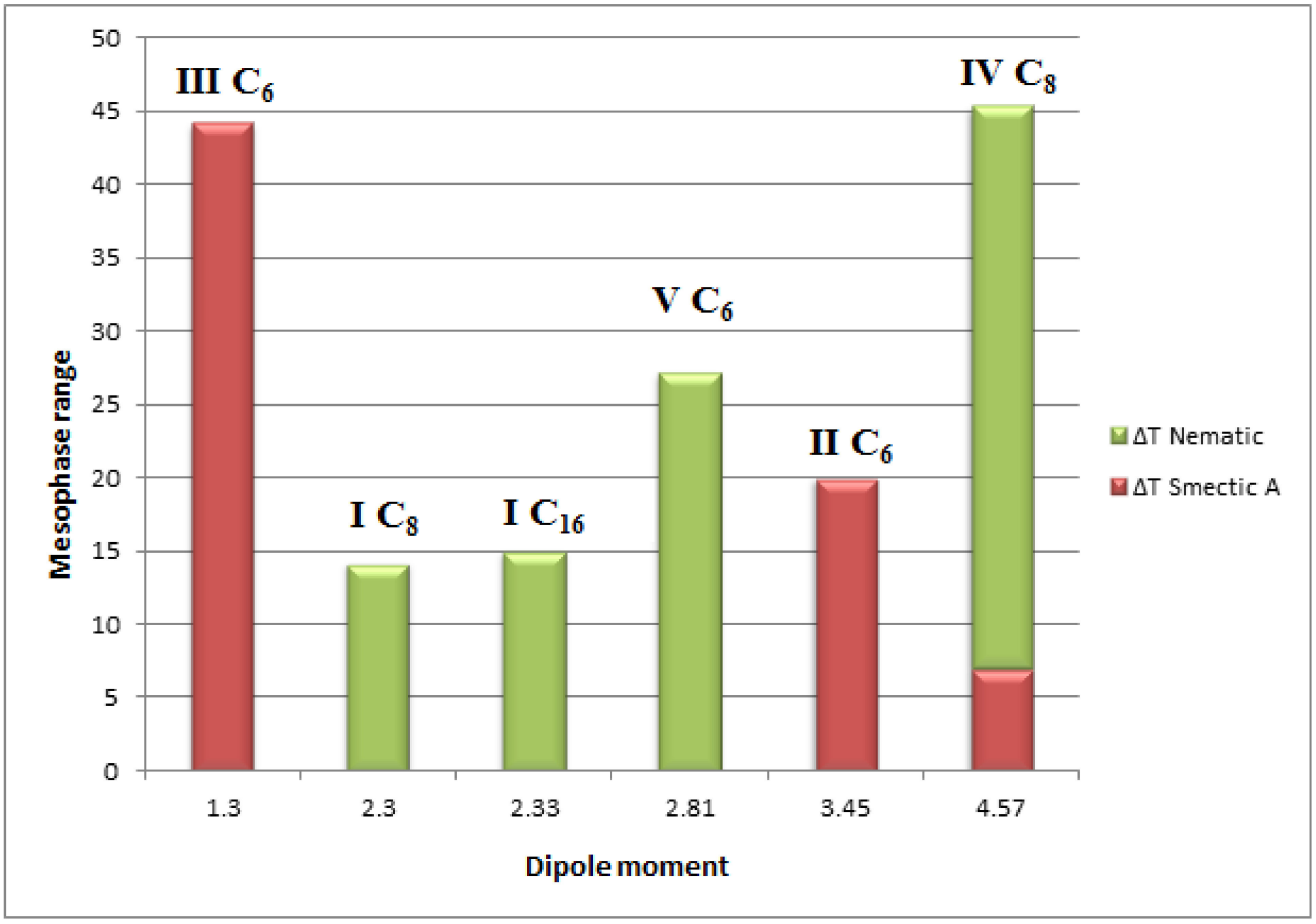
Graph of total mesophase range (nematic and smectic A mesophase) versus dipole moment of compounds **I C_8_**, **I C_16_**, **II C_6_**, **III C_6_**, **IV C_8_**, and **V C_6_**.

It is obvious from Figure 11 that the presence of nitrogen atoms decreased the dipole moment and mesophase range of the compounds **I C_8_** and **I C_16_**. However, the longer chain length (C = 16) increased the mesophase range without affecting its dipole moment. Its phenyl analog (**II C_6_**) showed a higher dipole moment with a longer mesophase range (ΔT = 20 °C). Moreover, the absence of nitrogen atoms permited the formation of smectic mesophase that could be explained in terms of the highly ordered liquid crystalline phase that neededa greater degree of packing. As previously discussed, the orientation of the mesogenic core (-CH=N-) highly impacted the dipole moment of the compound and it also affected the degree of packing to enhance smectic mesophase for the highly polar compound (**IV C_8_**). Moreover, the higher dipole moment (**IV C_8_**) influenced the mesophase range to be (ΔT = 45.3 °C) with respect to (ΔT = 27.2 °C) of its analogous compound (**V C_6_**). The presence of the terminal alkoxy group on the aniline ring of the compound (**III C_6_**) highly decreased the dipole moment. However, the compound showed a high mesophase range (ΔT = 44.2 °C). This result could be attributed to other structural factors that could share with the small dipole moment to show such a high mesophase range.

**Figure 12:**
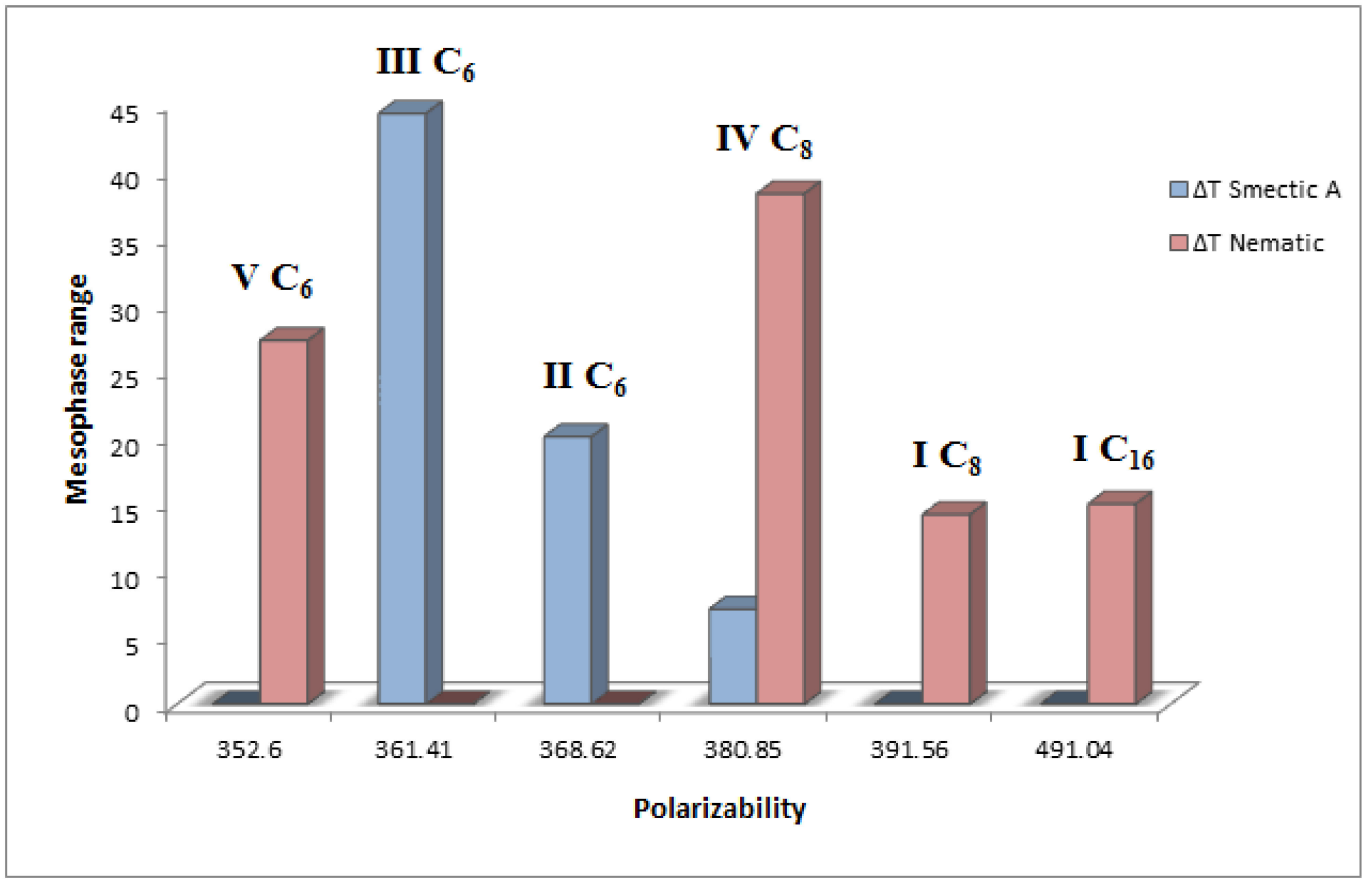
Graph of smectic A and nematic mesophase range versus polarizability of compounds **I C_8_**, **I C_16_**, **II C_6_**, **III C_6_**, **IV C_8_**, and **V C_6_**.

It is clear from Figure 12 that the polarizability of compound **III C_6_** was more than that of compound **V C_6_** with the same chain length (C=6), and such higher polarizability enhanced the mesophase range with the formation of smectic mesophase for compound **III C_6_**. On the other hand, the orientation of the mesogenic core highly affected the polarizability to show higher polarizability with a lower mesophase range. The orientation of the carboxylate group (COO) obviously had a high impact on both the polarizability and the mesomorphic behavior. Compound **II C_6_** had lower polarizability than compound **IV C_8_** with different mesomorphic behavior. Additionally, the presence of nitrogen atoms in the terminal ring of compound **I C_8_** increased the polarizability compared to its analogous compound **II C_6_** but it also decreased the mesophase range and the type of texture was changed from smectic A (**II C_6_**) to nematic (**I C_8_**).

#### 3.2.5. Aromaticity, LOL-π and π-π Stacking

**Figure 13:**
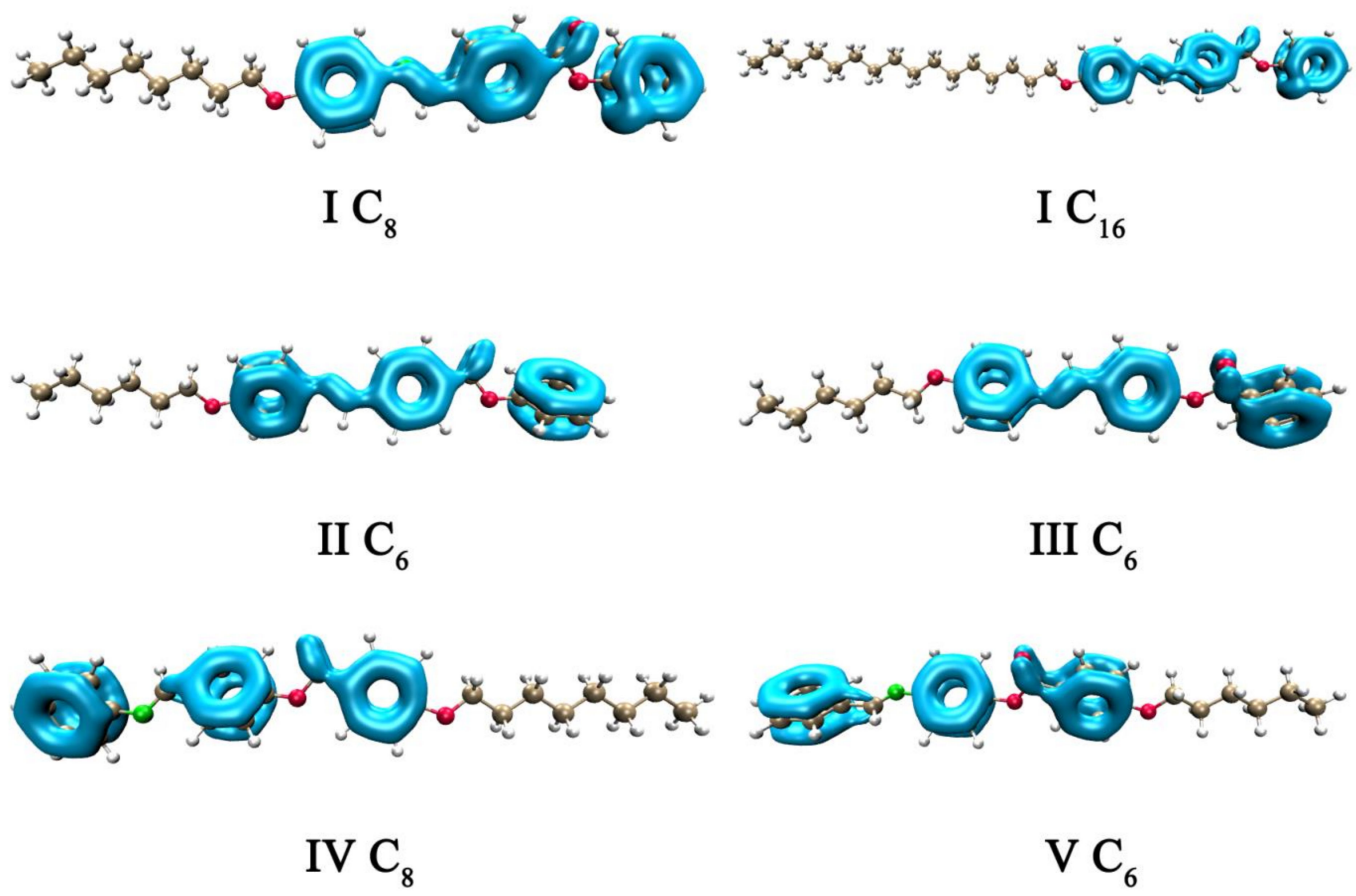
Isosurface maps of LOL- π (Isovalue = 0.3) of compounds **I C_8_**, **I C_16_**, **II C_6_**, **III C_6_**, **IV C_8_**, and **V C_6_**.

Multiwfn software in its 3.8 version[36] was used to study aromaticity, π-π stacking of all aromatic rings of the concerned compounds according to the optimized geometries obtained from Gaussian 09 software. Aromaticity was analyzed by the means of normalized multicenter bond order (MCBO index) which indicates the electron delocalization capability over a ring where a larger MCBO value refers to stronger aromaticity [37]. The extended π-π stacking was evaluated using the localized orbital locator integrated π over the plane (LOLIPOP index) where the higher LOLIPOP index value of a ring indicated a weaker π-stacking capability. The values of the MCBO index and the LOLIPOP index for all rings are tabulated in Table 6. Furthermore, the isosurface of the LOL purely contributed by π-orbitals (LOL-π) [38] was estimated using Multiwfn software then visualized using VMD software [39] to render high-quality figures as illustrated in Figure 13.

It was noticed from the normalized MCBO aromaticity index that A rings bounded to alkoxy chain in **I C_8_**, **I C_16_** and **II C_6_** have the same aromaticity (0.624). **III C_6_** had a slightly higher value (0.626) than the previously mentioned compounds. One could deduce that A rings in these compounds had almost the same degree of cyclic delocalization. Meanwhile, A rings of **IV C_8_** and **V C_6_** show an identical greater value (0.640) than other compounds which can be attributed to higher electron delocalization in the free benzene ring. Central B rings of **I C_8_**, **I C_16_** and **II C_6_** exhibited nearly similar aromatic character which was slightly lower than the aromaticity of **III C_6_** and **IV C_8_**. The B ring of **V C_6_** had the highest aromaticity index which may be because of a lack of extending π-conjugation outside the ring. C rings of **I C_8_** and **I C_16_** had identical aromaticity (0.643) which was lower than **II C_6_** (0.647) as the pyridine ring had a lower aromaticity character than benzene. It was observed that the C ring of **III C_6_** had a greater value than what **IV C_8_** and **V C_6_** had as the earlier one was free benzene.

It was revealed from the LOLIPOP index that inner benzene rings which are bounded to the alkoxy chain had stronger π-π stacking ability than terminal rings as seen in the C rings of **IV C_8_** and **V C_6_**, and also observed in the A rings of **II C_6_** and **III C_6_**. On the other hand, the C rings of **I C_8_** and **I C_16_** had the strongest π-π stacking ability which could be referred to as the presence of N atom.

It was found from LOL-π isosurfaces that benzene rings exert extending π-conjugation when bounded with the carbonyl group (C=O). It is also worth mentioning that the A ring in **IV C_8_** did not exhibit extending π-conjugation due to the electronegativity of the N atom thus, there was no resonance between the imine group and the benzene ring. However, the π-conjugation extended from the A ring to the carbon of the imine group in **V C_6_** which was probably due to the existence of resonance between the imine group and the benzene ring as the direction of electron-withdrawing was in line with the direction of resonance.

## 4. Conclusions

Herein liquid crystalline pyridyl analog has been prepared and fully characterized. The mesomorphic results of the prepared compound showed a nematic texture with a medium range. The investigated compounds were compared with respect to their phenyl analog to reveal that the phenyl analog has a smectic texture which could be attributed to its high dipole moment and low polarizability compared to what the pyridyl analog has. The energy gap between the FMO of pyridyl analog is lower than the energy gap of its phenyl analog which enhances the electronic transition. Moreover, the orientation of the mesogen showed a significant effect on the mesomorphic behavior where the CH=N and COO group’s orientation changed the texture of the investigated compounds from nematic to smectic or calamitic. Besides, it enhanced the mesomorphic range to 44.2 ^°^C for compound **III C_6_** instead of 20 ^°^C for compound **II C_6_**. Additionally, it developed a smectic mesophase in **IV C_8_** which did not exist in **V C_6_**. Twist angles in **I C_8_** and **I C_16_** compounds are lower than their analogous phenyl compound **II C_6_** which could indicate that the presence of nitrogen atom enhances the planarity which increases the mesophase temperature. In the aspect ratio approach, changing the position of the alkoxy terminal chain alters the aspect ratio significantly as seen in **III C_6_** and **V C_6_**, Additionally, changing the orientation of the mesogenic core (COO) in **II C_6_** and **III C_6_** may alter the aspect ratio value. The molecular electrostatic potential implied that the high degree of charge separation predicts high molecular packing which influences the mesophase ranges. It was found using LOLIPOP index values that the terminal ring of the pyridyl analog has a strong π-π stacking ability compared with its phenyl analog. Correspondingly in the phenyl analog case, the inner benzene rings which are bound to the alkoxy chain have a stronger π-π stacking ability than the terminal rings. Furthermore, the MCBO aromaticity index suggests that the free benzene rings which are not bound to the alkoxy chain have higher aromaticity than the inner benzene rings.

## Figures and Tables

**Table 2:** The estimated twist angles for compounds **I C_8_**, **I C_16_**, **II C_6_**, **III C_6_**, **IV C_8_**, and **V C_6_**.

Compounds	θ_A-B_	θ_A-C_	θ_B-C_
**I C_8_**	30.57	6.63	26.41
**I C_16_**	29.32	9.04	22.96
**II C_6_**	31.42	79.97	48.91
**III C_6_**	34.13	75.00	40.88
**IV C_8_**	43.78	12.21	31.59
**V C_6_**	41.00	89.63	48.63

**θ**_A-B_ = Twist angle between the planes of A ring and B ring; **θ**_A-C_ = Twist angle between the planes of A ring and C ring; **θ**_B-C_ = Twist angle between the planes of B ring and C ring.

**Table 3:** The calculated dimensions (A), area (A^2^), and aspect ratio of compounds **I C_8_**, **I C_16_**, **II C_6_**, **III C_6_**, **IV C_8_**, and **V C_6_**.

Compounds	Length	Width	Height	Area	Aspect Ratio
**I C_8_**	31.35	7.48	4.63	234.50	4.19
**I C_16_**	41.60	7.74	4.83	321.98	5.38
**II C_6_**	28.78	7.27	6.11	209.23	3.96
**III C_6_**	28.66	7.47	5.97	214.09	3.84
**IV C_8_**	31.52	7.50	4.72	236.40	4.20
**V C_6_**	28.89	6.92	6.02	199.92	4.18

**Table 4:** The energies (eV), molecular orbitals energies (eV) of compounds **I C_8_**, **I C_16_**, **II C_6_**, **III C_6_**, **IV C_8_**, and **V C_6_**.

Compounds	Energy	E_LUMO_	E_HOMO_	ΔE_(LUMO-HOMO)_
**I C_8_**	−37619.24	−2.30	−5.80	3.50
**I C_16_**	−46179.77	−2.30	−5.80	3.50
**II C_6_**	−35042.51	−2.28	−5.81	3.53
**III C_6_**	−35042.52	−1.88	−5.61	3.73
**IV C_8_**	−37182.74	−1.83	−6.03	4.20
**V C_6_**	−35042.58	−1.81	−5.98	4.17

**Table 5:** Polarizability α (a_0_^3^) and dipole moments (debye) of compounds **I C_8_**, **I C_16_**, **II C_6_**, **III C_6_**, **IV C_8_**, and **V C_6_**.

Compounds		I C_8_	I C_16_	II C_6_	III C_6_	IV C_8_	V C_6_
**Polarizability**		391.56	491.04	368.62	361.41	380.85	352.60
**Dipole moment**	**(x)**	−1.89	1.98	−2.87	0.07	4.52	−2.36
	**(y)**	−0.14	−0.12	−1.86	1.14	0.29	−1.40
	**(z)**	−1.31	1.23	0.51	0.61	−0.60	0.59
	**(Total)**	2.30	2.33	3.45	1.30	4.57	2.81

**Table 6:** The normalized multicenter bond order (MCBO) index and localized orbital locator integrated π over the plane (LOLIPOP) of compounds **I C_8_**, **I C_16_**, **II C_6_**, **III C_6_**, **IV C_8_**, and **V C_6_**.

Compounds	MCBO Index			LOLIPOP Index		
	A	B	C	A	B	C
**I C_8_**	0.624	0.629	0.643	6.751	6.691	5.041
**I C_16_**	0.624	0.629	0.643	6.740	6.703	5.055
**II C_6_**	0.624	0.630	0.647	6.741	6.685	7.253
**III C_6_**	0.626	0.632	0.643	6.814	6.777	7.135
**IV C_8_**	0.640	0.632	0.626	7.275	6.954	7.033
**V C_6_**	0.640	0.634	0.626	7.171	7.021	7.031

## Data Availability

Not applicable.

## Supplementary Materials

The following supporting information can be downloaded at: https://www.mdpi.com/article/10.3390/molecules27072304/s1, Figure S1: 1HNMR of Pyridin-2-yl 4-[4-(hexyloxy)phenyliminomethyl]benzoate (I C6); Figure S2: 1HNMR of Pyridin-2-yl 4-[4-(hexadecyloxy)phenyliminomethyl]benzoate (I C16).

## Sample Availability Statement

Samples of the compounds are available from the authors.

## References

[1] Dierking I., Al-Zangana S. (2017). Lyotropic liquid crystal phases from anisotropic nanomaterials. Nanomaterials.

[2] Lagerwall J.P., Scalia G. (2012). A new era for liquid crystal research: Applications of liquid crystals in soft matter nano-, bio- and microtechnology. Curr. Appl. Phys..

[3] An J.-G., Hina S., Yang Y., Xue M., Liu Y. (2016). Characterization of liquid crystals: A literature review. Rev. Adv. Mater. Sci..

[4] Gray G.W., Vill V., Spiess H.W., Demus D., Goodby J.W. (2009). Physical Properties of Liquid Crystals.

[5] Kumar S. (2001). Experimental Study of Physical Properties and Phase Transitions.

[6] Zhang M., Jang C.-H. (2013). Liquid crystal based optical sensor for imaging trypsin activity at interfaces between aqueous phases and thermotropic liquid crystals. Bull. Korean Chem. Soc..

[7] Sergeyev S., Pisula W., Geerts Y.H. (2007). Discotic liquid crystals: A new generation of organic semiconductors. Chem. Soc. Rev..

[8] O’Neill M., Kelly S.M. (2011). Ordered materials for organic electronics and photonics. Adv. Mater..

[9] Yu Y., Nakano M., Ikeda T. (2003). Directed bending of a polymer film by light. Nature.

[10] Thomsen D.L., Keller P., Naciri J., Pink R., Jeon H., Shenoy D., Ratna B.R. (2001). Liquid crystal elastomers with mechanical properties of a muscle. Macromolecules.

[11] Rananavare S.B., Pisipati V., Iwan A., Schab-Balcerzak E. (2011). An Overview of Liquid Crystals Based on Schiff Base Compounds. Liquid Crystalline Organic Compounds and Polymers as Materials of the XXI Century: From Synthesis to Applications.

[12] Kelker H., Scheurle B. (1969). A liquid-crystalline (nematic) phase with a particularly low solidification point. Angew. Chem. Int. Ed. Engl..

[13] Hallsby A., Nilsson M., Otterholm B. (1982). Synthesis of Schiff Bases Forming the First Room Temperature Ferroelectric Liquid Crystal—The Mora Series. Mol. Cryst. Liq. Cryst..

[14] Gomha S.M., Ahmed H.A., Shaban M., Abolibda T.Z., Khushaim M.S., Alharbi K.A. (2021). Synthesis, optical characterizations and solar energy applications of new Schiff base materials. Materials.

[15] Wu J.-N., Chen L., Fu T., Zhao H.-B., Guo D.-M., Wang X.-L., Wang Y.-Z. (2018). New application for aromatic Schiff base: High efficient flame-retardant and anti-dripping action for polyesters. Chem. Eng. J..

[16] Yeap G.-Y., Ha S.-T., Boey P.-L., Mahmood W.A.K., Ito M.M., Youhei Y. (2006). Synthesis and characterization of some new mesogenic schief base esters N-[4-(4-n-hexadecanoyloxybenzoyloxy)-benzylidene]-4-substituted anilines. Mol. Cryst. Liq. Cryst..

[17] Ahmed H., Hagar M., El-Sayed T., Alnoman R.B. (2019). Schiff base/ester liquid crystals with different lateral substituents: Mesophase behaviour and DFT calculations. Liq. Cryst..

[18] Ahmed H., Hagar M., Saad G. (2019). Impact of the proportionation of dialkoxy chain length on the mesophase behaviour of Schiff base/ester liquid crystals; experimental and theoretical study. Liq. Cryst..

[19] Alnoman R., Ahmed H.A., Hagar M. (2019). Synthesis, optical, and geometrical approaches of new natural fatty acids’ esters/Schiff base liquid crystals. Molecules.

[20] Nafee S.S., Hagar M., Ahmed H.A., Alhaddad O., El-Shishtawy R.M., Raffah B.M. (2020). New two rings Schiff base liquid crystals; ball mill synthesis, mesomorphic, Hammett and DFT studies. J. Mol. Liq..

[21] Ahmed N.H., Saad G.R., Ahmed H.A., Hagar M. (2020). New wide-stability four-ring azo/ester/Schiff base liquid crystals: Synthesis, mesomorphic, photophysical, and DFT approaches. RSC Adv..

[22] Hagar M., Ahmed H., Saad G. (2020). New calamitic thermotropic liquid crystals of 2-hydroxypyridine ester mesogenic core: Mesophase behaviour and DFT calculations. Liq. Cryst..

[23] Nafee S.S., Hagar M., Ahmed H.A., El-Shishtawy R.M., Raffah B.M. (2019). The synthesis of new thermal stable schiff base/ester liquid crystals: A computational, mesomorphic, and optical study. Molecules.

[24] Hagar M., Ahmed H., Aouad M. (2020). Mesomorphic and DFT diversity of Schiff base derivatives bearing protruded methoxy groups. Liq. Cryst..

[25] Hagar M., Ahmed H., Saad G. (2018). Mesophase stability of new Schiff base ester liquid crystals with different polar substituents. Liq. Cryst..

[26] Hagar M., Ahmed H., Saad G. (2019). Synthesis and mesophase behaviour of Schiff base/ester 4-(arylideneamino) phenyl-4″-alkoxy benzoates and their binary mixtures. J. Mol. Liq..

[27] Yamamura Y., Adachi T., Miyazawa T., Horiuchi K., Sumita M., Massalska-Arodź M., Urban S., Saito K. (2012). Calorimetric and spectroscopic evidence of chain-melting in smectic e and smectic a phases of 4-alkyl-4′-isothiocyanatobiphenyl (n TCB). J. Phys. Chem. B.

[28] Wunderlich B. (1999). A classification of molecules, phases, and transitions as recognized by thermal analysis. Thermochim. Acta.

[29] Frisch M., Trucks G., Schlegel H.B., Scuseria G.E., Robb M.A., Cheeseman J.R., Scalmani G., Barone V., Mennucci B., Petersson G. (2009). Gaussian 09, Revision D. 01.

[30] Romeo M. (2021). Density–orientation coupling for a microcontinuum approach to nematic liquid crystals subject to electric field. Contin. Mech. Thermodyn..

[31] Chen R., Wang L., An Z., Chen X., Chen P. (2021). Effect of π-conjugation units on the liquid crystal and photovoltaic performance of heterocyclic pyridine-based compounds. Liq. Cryst..

[32] Li J., Xia R., Xu H., Yang J., Zhang X., Kougo J., Lei H., Dai S., Huang H., Zhang G. (2021). How Far Can We Push the Rigid Oligomers/Polymers toward Ferroelectric Nematic Liquid Crystals?. J. Am. Chem. Soc..

[33] Jain V., Kaur S., Mohiuddin G., Pal S.K. (2021). Design, Synthesis and Characterization of Achiral Unsymmetrical Four-Ring Based Hockey-Stick Shaped Liquid Crystals: Structure-Property Relationship. Liq. Cryst..

[34] Goodby J.W. (2017). Free volume, molecular grains, self-organisation, and anisotropic entropy: Machining materials. Liq. Cryst..

[35] Singh S. (2002). Liquid Crystals: Fundamentals.

[36] Lu T., Chen F. (2012). Multiwfn: A multifunctional wavefunction analyzer. J. Comput. Chem..

[37] Giambiagi M., de Giambiagi M.S., Mundim K.C. (1990). Definition of a multicenter bond index. Struct. Chem..

[38] Lu T., Chen Q. (2020). A simple method of identifying π orbitals for non-planar systems and a protocol of studying π electronic structure. Theor. Chem. Acc..

[39] Humphrey W., Dalke A., Schulten K. (1996). VMD: Visual molecular dynamics. J. Mol. Graph..

