# Supplementary material for: Three Rings Schiff Base Ester Liquid Crystals: Experimental and Computational Approaches of Mesogenic Core Orientation Effect, Heterocycle Impact
Source: Molecules. 2022 Apr 1;27(7):2304. doi: 10.3390/molecules27072304 (PMC9000867; doi:10.3390/molecules27072304)
Supplement: Supplementary file 1 [file molecules-27-02304-s001.zip › molecules-1647434-supplementary.pdf]

Supplementary Material

Three Rings Schiff Base Ester Liquid Crystals: Experimental and Computational Approaches of Mesogenic Core Orientation Effect, Heterocycle Impact

Shady Nada [1], Mohamed Hagar [1,*] Omaima Farahat [1], Ahmed A. Hasanein [1], Abdul−Hamid Emwas [2], Abeer Ali Sharfalddin [3], Mariusz Jaremko [4] and Mohamed A. Zakaria [1]

[1] Department of Chemistry, Faculty of Science, Alexandria University, Alexandria 21321, Egypt; shadyadel4869@gmail.com (S.N.); oom_farahat@yahoo.com (O.F.); ahmedhasanein@alexu.edu.eg (A.A.H.); mohamed.zakaria@alexu.edu.eg (M.A.Z.)

[2] Core Labs, King Abdullah University of Science and Technology, P.O. Box 4700, Thuwal 23955−6900, Saudi Arabia; abdelhamid.emwas@kaust.edu.sa

[3] Department of Chemistry, Faculty of Science, King Abdulaziz University, P.O. Box 80203, Jeddah 21589, Saudi Arabia; sharfalddin.aa@hotmail.com

[4] Smart−Health Initiative (SHI) and Red Sea Research Center (RSRC), Division of Biological and Environmental Sciences and Engineering (BESE), King Abdullah University of Science and Technology (KAUST), P.O.Box 4700, Thuwal 23955−6900, Saudi Arabia; mariusz.jaremko@kaust.edu.sa

* Correspondence: mohamed.hagar@alexu.edu.eg

Coordinates of the investigated compounds

I C$_{8}$ Compound (in Angstrom Å)

6	−14.776023000	0.223987000	−0.181545000
6	−13.406316000	0.893647000	−0.323927000
6	−12.234797000	−0.071373000	−0.107762000
6	−10.859640000	0.591717000	−0.243317000
6	−9.689048000	−0.372789000	−0.021911000
6	−8.315198000	0.295553000	−0.148801000
6	−7.149656000	−0.672468000	0.083391000
6	−5.795299000	0.008629000	−0.029586000
8	−4.789664000	−0.978154000	0.213116000
6	−3.480761000	−0.607280000	0.200313000
6	−2.556301000	−1.620505000	0.493294000
6	−1.199469000	−1.351931000	0.505991000
6	−0.718559000	−0.057200000	0.230843000
6	−1.653197000	0.951484000	−0.024757000
6	−3.018797000	0.687172000	−0.059957000
7	0.635816000	0.308342000	0.240066000
6	1.556356000	−0.532210000	−0.038275000
6	2.979672000	−0.179405000	0.019620000
6	3.943591000	−1.139512000	−0.318140000
6	5.298104000	−0.836243000	−0.271419000
6	5.709900000	0.443456000	0.115506000
6	4.750105000	1.408693000	0.456753000
6	3.402211000	1.102438000	0.410393000
6	7.142105000	0.834600000	0.190935000

8	7.541392000	1.912089000	0.553611000
8	7.948458000	−0.197253000	−0.204615000
6	9.336432000	−0.144818000	−0.205893000
7	9.873345000	−1.341199000	−0.043460000
6	11.205802000	−1.433411000	−0.082394000
6	12.042040000	−0.341532000	−0.285575000
6	11.451135000	0.907754000	−0.458571000
6	10.066952000	1.025943000	−0.422931000
1	−15.588862000	0.937048000	−0.343857000
1	−14.904708000	−0.204367000	0.817424000
1	−14.896626000	−0.587486000	−0.906084000
1	−13.327910000	1.720914000	0.391862000
1	−13.322106000	1.345254000	−1.319849000
1	−12.311425000	−0.898291000	−0.825676000
1	−12.321331000	−0.525639000	0.887870000
1	−10.785766000	1.419854000	0.473348000
1	−10.772592000	1.044007000	−1.239682000
1	−9.757473000	−1.198443000	−0.741397000
1	−9.777782000	−0.827952000	0.972603000
1	−8.251127000	1.123762000	0.568510000
1	−8.224430000	0.746846000	−1.145082000
1	−7.190268000	−1.492731000	−0.640910000
1	−7.228917000	−1.125067000	1.077357000
1	−5.705532000	0.818047000	0.706282000
1	−5.658634000	0.440339000	−1.029562000
1	−2.934895000	−2.609214000	0.723011000

1	−0.507426000	−2.143669000	0.768278000
1	−1.284818000	1.953306000	−0.210224000
1	−3.708163000	1.491696000	−0.276566000
1	1.337248000	−1.562161000	−0.350042000
1	3.626084000	−2.132957000	−0.617680000
1	6.039487000	−1.581300000	−0.526185000
1	5.088627000	2.392425000	0.757222000
1	2.650768000	1.836354000	0.672181000
1	11.614726000	−2.429951000	0.055591000
1	13.117370000	−0.467332000	−0.307341000
1	12.059899000	1.789672000	−0.622065000
1	9.572894000	1.977187000	−0.536150000

I C_{16} Compound (in Angstrom Å)

6	10.278185000	−0.449050000	0.027183000
6	8.938648000	0.296357000	0.021592000
6	7.721033000	−0.631424000	−0.058842000
6	6.380243000	0.111714000	−0.058166000
6	5.165664000	−0.818486000	−0.151802000
6	3.823625000	−0.077351000	−0.140087000
6	2.617592000	−1.016450000	−0.253602000
6	1.291759000	−0.273256000	−0.222566000
8	0.248591000	−1.241119000	−0.361275000
6	−1.046701000	−0.825723000	−0.362417000

6	−2.009189000	−1.830218000	−0.541457000
6	−3.356742000	−1.518877000	−0.552076000
6	−3.790421000	−0.189051000	−0.388089000
6	−2.818715000	0.807419000	−0.249800000
6	−1.461391000	0.502382000	−0.218783000
7	−5.132736000	0.219136000	−0.394029000
6	−6.070195000	−0.565394000	−0.023468000
6	−7.482870000	−0.170795000	−0.065457000
6	−8.464133000	−1.072756000	0.368960000
6	−9.809265000	−0.728243000	0.345116000
6	−10.194142000	0.535009000	−0.116408000
6	−9.217142000	1.441496000	−0.555937000
6	−7.878571000	1.094201000	−0.531830000
6	−11.615189000	0.968806000	−0.168186000
8	−11.993249000	2.031148000	−0.593412000
8	−12.436095000	−0.004127000	0.331401000
6	−13.820382000	0.086889000	0.392759000
7	−14.386054000	−1.107620000	0.410869000
6	−15.716111000	−1.163027000	0.522284000
6	−16.522810000	−0.034949000	0.621395000
6	−15.902425000	1.211700000	0.603939000
6	−14.519591000	1.292689000	0.489158000
1	10.349086000	−1.071117000	−0.874473000
1	10.301705000	−1.143600000	0.876814000
1	8.917049000	0.995172000	−0.824577000
1	8.864523000	0.913497000	0.926361000

1	7.745135000	−1.333844000	0.784204000
1	7.793652000	−1.244671000	−0.966320000
1	6.358597000	0.820128000	−0.896264000
1	6.303217000	0.717812000	0.853766000
1	5.190281000	−1.532447000	0.681067000
1	5.238444000	−1.417873000	−1.068115000
1	3.803622000	0.645314000	−0.965725000
1	3.745662000	0.511088000	0.783101000
1	2.628440000	−1.744293000	0.564425000
1	2.673090000	−1.588689000	−1.185564000
1	1.233756000	0.454362000	−1.042340000
1	1.172485000	0.269098000	0.724178000
1	−1.667184000	−2.848205000	−0.684204000
1	−4.078673000	−2.308536000	−0.724547000
1	−3.151224000	1.833803000	−0.151189000
1	−0.742466000	1.300502000	−0.094076000
1	−5.873916000	−1.574319000	0.363125000
1	−8.167472000	−2.053151000	0.727152000
1	−10.564208000	−1.428297000	0.676243000
1	−9.534828000	2.413242000	−0.912885000
1	−7.114161000	1.783216000	−0.867699000
1	−16.148263000	−2.159288000	0.532519000
1	−17.597783000	−0.131838000	0.708975000
1	−16.486961000	2.121453000	0.680121000
1	−14.004201000	2.238535000	0.455460000
6	11.497615000	0.477125000	0.098020000

1	11.475236000	1.169780000	−0.753207000
1	11.426387000	1.101163000	0.998297000
6	12.836264000	−0.269870000	0.106119000
1	12.905071000	−0.897381000	−0.791937000
1	12.860296000	−0.959437000	0.959808000
6	14.056785000	0.655393000	0.169665000
1	14.032775000	1.344354000	−0.684525000
1	13.988374000	1.283504000	1.067331000
6	15.395002000	−0.092379000	0.177543000
1	15.461744000	−0.722773000	−0.718642000
1	15.420407000	−0.779235000	1.033377000
6	16.616007000	0.832619000	0.235841000
1	16.589960000	1.519735000	−0.619806000
1	16.549970000	1.462742000	1.132302000
6	17.954190000	0.085167000	0.242032000
1	18.020613000	−0.546080000	−0.653773000
1	17.982164000	−0.601167000	1.098420000
6	19.175745000	1.009661000	0.297777000
1	19.147736000	1.695492000	−0.557650000
1	19.110480000	1.639527000	1.193341000
6	20.507974000	0.254471000	0.302204000
1	20.619591000	−0.356032000	−0.599396000
1	21.357483000	0.941684000	0.344511000
1	20.579787000	−0.415550000	1.164743000

II C_6 Compound (in Angstrom Å)

6	−13.283298000	−0.033812000	0.526323000
6	−11.910672000	0.619004000	0.712640000
6	−10.756278000	−0.240867000	0.185894000
6	−9.377051000	0.400487000	0.376933000
6	−8.231924000	−0.464379000	−0.161496000
6	−6.867248000	0.169755000	0.054821000
8	−5.886670000	−0.714074000	−0.493928000
6	−4.570268000	−0.382439000	−0.404439000
6	−3.671675000	−1.289085000	−0.985409000
6	−2.309201000	−1.053883000	−0.941890000
6	−1.796976000	0.100883000	−0.319819000
6	−2.706328000	1.017087000	0.218640000
6	−4.077272000	0.780081000	0.197519000
7	−0.433737000	0.422157000	−0.236667000
6	0.463088000	−0.486402000	−0.198766000
6	1.895497000	−0.170231000	−0.156097000
6	2.830555000	−1.211904000	−0.082693000
6	4.192542000	−0.943844000	−0.032912000
6	4.642565000	0.380051000	−0.058151000
6	3.711407000	1.426921000	−0.134608000
6	2.355524000	1.156947000	−0.182975000
6	6.085257000	0.740269000	−0.007067000
8	6.871241000	−0.375729000	0.107598000
8	6.515259000	1.863051000	−0.054947000

6	8.261909000	−0.276533000	0.184592000
6	8.875203000	−1.049381000	1.164960000
6	10.264466000	−1.065455000	1.253424000
6	11.031114000	−0.311023000	0.367947000
6	10.400910000	0.456771000	−0.609399000
6	9.011927000	0.479327000	−0.711740000
1	−14.084091000	0.603696000	0.910497000
1	−13.340086000	−0.991718000	1.052531000
1	−13.489936000	−0.225447000	−0.531219000
1	−11.747740000	0.828946000	1.776727000
1	−11.896880000	1.591343000	0.205561000
1	−10.915528000	−0.445753000	−0.880457000
1	−10.773246000	−1.216288000	0.688468000
1	−9.217286000	0.597662000	1.444744000
1	−9.362579000	1.378811000	−0.120033000
1	−8.366941000	−0.642986000	−1.233380000
1	−8.239868000	−1.445385000	0.325011000
1	−6.674447000	0.320277000	1.124808000
1	−6.807067000	1.146001000	−0.443388000
1	−4.074346000	−2.166669000	−1.476684000
1	−1.637045000	−1.753115000	−1.425399000
1	−2.313969000	1.919469000	0.671960000
1	−4.746582000	1.505899000	0.638663000
1	0.214625000	−1.555993000	−0.177594000
1	2.483805000	−2.240038000	−0.062740000
1	4.909286000	−1.751587000	0.024821000

1	4.078883000	2.445334000	−0.154759000
1	1.626704000	1.955292000	−0.240997000
1	8.258538000	−1.627619000	1.842133000
1	10.745011000	−1.667417000	2.016099000
1	12.112516000	−0.321399000	0.438200000
1	10.991759000	1.045809000	−1.301502000
1	8.521388000	1.081710000	−1.462589000

III C_6 Compound (in Angstrom Å)

6	−13.272839000	0.437612000	−0.382742000
6	−11.862790000	0.956626000	−0.677551000
6	−10.756670000	0.058760000	−0.111997000
6	−9.342447000	0.575498000	−0.398917000
6	−8.241992000	−0.325160000	0.172814000
6	−6.846502000	0.207532000	−0.110283000
8	−5.907644000	−0.699512000	0.469394000
6	−4.576462000	−0.425533000	0.369917000
6	−3.715936000	−1.333939000	1.001040000
6	−2.344588000	−1.149165000	0.959013000
6	−1.785640000	−0.045708000	0.287338000
6	−2.657265000	0.871744000	−0.307540000
6	−4.036266000	0.684469000	−0.287599000
7	−0.409582000	0.224275000	0.215896000
6	0.448950000	−0.720945000	0.193736000

6	1.893632000	−0.471596000	0.176254000
6	2.785784000	−1.549446000	0.122531000
6	4.163031000	−1.353353000	0.103914000
6	4.651400000	−0.050072000	0.135650000
6	3.782308000	1.041213000	0.192002000
6	2.414139000	0.831593000	0.213393000
8	6.005868000	0.264278000	0.198809000
6	6.950816000	−0.412433000	−0.527166000
8	6.700020000	−1.315000000	−1.282080000
6	8.315514000	0.119138000	−0.260659000
6	9.380964000	−0.458873000	−0.960590000
6	10.678267000	−0.006957000	−0.752722000
6	10.920086000	1.023143000	0.156065000
6	9.861791000	1.600736000	0.856051000
6	8.560879000	1.153098000	0.650260000
1	−14.037072000	1.099532000	−0.798885000
1	−13.449488000	0.362865000	0.694776000
1	−13.426313000	−0.557519000	−0.811816000
1	−11.752398000	1.967076000	−0.265506000
1	−11.728769000	1.054661000	−1.761665000
1	−10.864135000	−0.951950000	−0.526073000
1	−10.891883000	−0.041569000	0.972497000
1	−9.238760000	1.586877000	0.014731000
1	−9.208312000	0.674405000	−1.483714000
1	−8.322860000	−1.333034000	−0.247738000
1	−8.362471000	−0.425279000	1.256623000

1	−6.716411000	1.206211000	0.326754000
1	−6.671592000	0.284324000	−1.191433000
1	−4.152843000	−2.171374000	1.531755000
1	−1.700628000	−1.846237000	1.482726000
1	−2.230287000	1.736025000	−0.802136000
1	−4.674907000	1.409690000	−0.773256000
1	0.152181000	−1.778729000	0.171990000
1	2.397653000	−2.562508000	0.094370000
1	4.839628000	−2.192512000	0.049871000
1	4.198974000	2.040656000	0.220810000
1	1.723445000	1.664114000	0.257148000
1	9.169815000	−1.258022000	−1.660013000
1	11.500888000	−0.456187000	−1.296994000
1	11.932588000	1.375413000	0.318672000
1	10.050686000	2.400713000	1.562600000
1	7.735405000	1.596386000	1.190364000

IV C_8 Compound (in Angstrom Å)

6	11.980655000	−0.589587000	−0.003007000
6	13.253359000	0.264373000	0.031282000
6	10.687607000	0.232348000	0.040314000
6	9.416495000	−0.623197000	−0.000202000
6	14.539810000	−0.565263000	−0.010073000
6	8.124073000	0.200024000	0.050915000

6	6.858669000	−0.663142000	−0.008180000
6	5.585794000	0.164619000	0.058471000
8	4.475735000	−0.737437000	−0.031864000
6	3.213815000	−0.247257000	−0.002744000
6	2.888903000	1.108911000	0.136180000
6	1.554775000	1.494926000	0.152566000
6	0.528419000	0.554311000	0.030618000
6	0.864076000	−0.802391000	−0.108288000
6	2.188063000	−1.198029000	−0.124061000
6	−0.866142000	1.047697000	0.054688000
8	−1.192320000	2.200950000	0.177341000
8	−1.759054000	0.010363000	−0.078884000
6	−3.135093000	0.186073000	−0.108919000
6	−3.776827000	1.297238000	−0.653018000
6	−5.167854000	1.313044000	−0.684294000
6	−5.925651000	0.245562000	−0.189602000
6	−5.254648000	−0.863517000	0.349452000
6	−3.872188000	−0.892644000	0.389419000
6	−7.388728000	0.304665000	−0.247048000
7	−8.135112000	−0.652743000	0.147683000
6	−9.533633000	−0.519353000	0.121679000
6	−10.200252000	0.656224000	0.501416000
6	−11.590811000	0.713972000	0.476918000
6	−12.336164000	−0.391295000	0.072058000
6	−11.679392000	−1.567507000	−0.291483000
6	−10.292371000	−1.638357000	−0.251395000

1	11.993352000	−1.289689000	0.842382000
1	11.986321000	−1.208976000	−0.909317000
1	13.247160000	0.884081000	0.936173000
1	13.241712000	0.962447000	−0.814509000
1	10.677492000	0.934997000	−0.802852000
1	10.680964000	0.848251000	0.948859000
1	9.427377000	−1.329656000	0.839264000
1	9.419430000	−1.233988000	−0.911766000
1	15.427715000	0.072221000	0.016223000
1	14.591663000	−1.169266000	−0.921348000
1	14.596791000	−1.249196000	0.842508000
1	8.118885000	0.914151000	−0.782312000
1	8.116502000	0.801353000	0.968950000
1	6.852391000	−1.380596000	0.818924000
1	6.845396000	−1.248019000	−0.933734000
1	5.543333000	0.883172000	−0.769335000
1	5.532369000	0.722860000	1.001250000
1	3.660958000	1.859776000	0.230835000
1	1.289823000	2.539410000	0.260643000
1	0.079058000	−1.539882000	−0.205541000
1	2.464443000	−2.239629000	−0.231066000
1	−3.206475000	2.132916000	−1.025919000
1	−5.673701000	2.176167000	−1.104929000
1	−5.840447000	−1.690308000	0.730988000
1	−3.339393000	−1.740829000	0.801629000
1	−7.809651000	1.227484000	−0.670361000

1	−9.627496000	1.508243000	0.849458000
1	−12.093586000	1.624503000	0.784204000
1	−13.418871000	−0.342877000	0.055458000
1	−12.252052000	−2.436271000	−0.596889000
1	−9.769110000	−2.549351000	−0.516374000

V C_6 Compound (in Angstrom Å)

6	−3.879359000	0.854396000	0.518413000
6	−4.275160000	−0.251782000	−0.244900000
6	−3.302271000	−1.053472000	−0.862175000
6	−1.959756000	−0.757220000	−0.718212000
6	−1.553410000	0.349056000	0.044957000
6	−2.527268000	1.143310000	0.655123000
6	−0.133824000	0.723896000	0.236943000
8	−5.561328000	−0.628425000	−0.446229000
6	−6.620196000	0.131598000	0.148090000
6	−7.933731000	−0.525640000	−0.242803000
6	−9.151288000	0.202604000	0.337422000
6	−10.483089000	−0.452840000	−0.045671000
6	−11.707601000	0.267858000	0.529275000
6	−13.033767000	−0.393373000	0.143600000
8	0.254214000	1.674680000	0.866472000
8	0.704497000	−0.159813000	−0.398265000
6	2.087490000	0.008122000	−0.359231000

6	2.840321000	−1.133970000	−0.109448000
6	4.228760000	−1.067054000	−0.132206000
6	4.877376000	0.146078000	−0.408072000
6	4.096333000	1.275270000	−0.693517000
6	2.709985000	1.219204000	−0.656461000
7	6.273839000	0.282819000	−0.470520000
6	7.023766000	−0.337741000	0.354769000
6	8.489133000	−0.275117000	0.296549000
6	9.246426000	−0.955020000	1.259068000
6	10.637319000	−0.909969000	1.227217000
6	11.285202000	−0.183234000	0.231264000
6	10.537817000	0.498216000	−0.733062000
6	9.151400000	0.453733000	−0.703650000
1	−4.609997000	1.487393000	1.002399000
1	−3.633366000	−1.902065000	−1.447976000
1	−1.214102000	−1.377839000	−1.196489000
1	−2.206727000	1.995715000	1.241288000
1	−6.579227000	1.166785000	−0.212899000
1	−6.499226000	0.143093000	1.238435000
1	−7.918144000	−1.565519000	0.099881000
1	−7.997473000	−0.554474000	−1.335526000
1	−9.152085000	1.245581000	−0.004122000
1	−9.069751000	0.239284000	1.431281000
1	−10.483862000	−1.495873000	0.295569000
1	−10.566150000	−0.490892000	−1.139309000
1	−11.706167000	1.309989000	0.187468000

1	−11.622964000	0.306630000	1.622046000
1	−13.080240000	−1.426079000	0.502948000
1	−13.163492000	−0.415855000	−0.942912000
1	2.329260000	−2.068540000	0.087897000
1	4.810876000	−1.966520000	0.030071000
1	4.601960000	2.202577000	−0.933686000
1	2.119089000	2.102156000	−0.852895000
1	6.607270000	−0.945579000	1.170278000
1	8.739831000	−1.519800000	2.035409000
1	11.213886000	−1.439336000	1.977238000
1	12.368489000	−0.146329000	0.203926000
1	11.043228000	1.063550000	−1.508052000
1	8.555372000	0.975132000	−1.442328000
1	−13.885882000	0.144085000	0.568183000

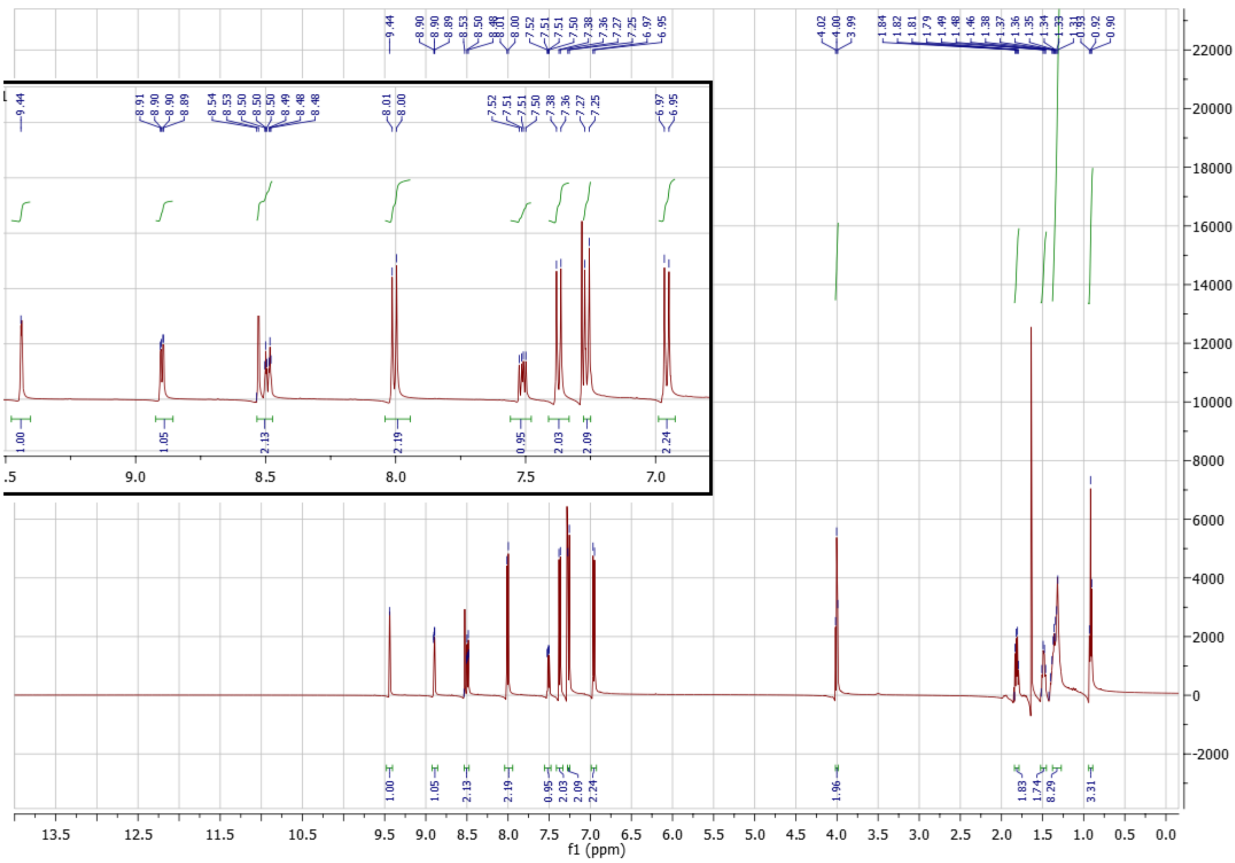

Figure S1. ^{1}HNMR of Pyridin−2−yl 4−[4−(octyloxy)phenyliminomethyl]benzoate (**I C**$_8$).

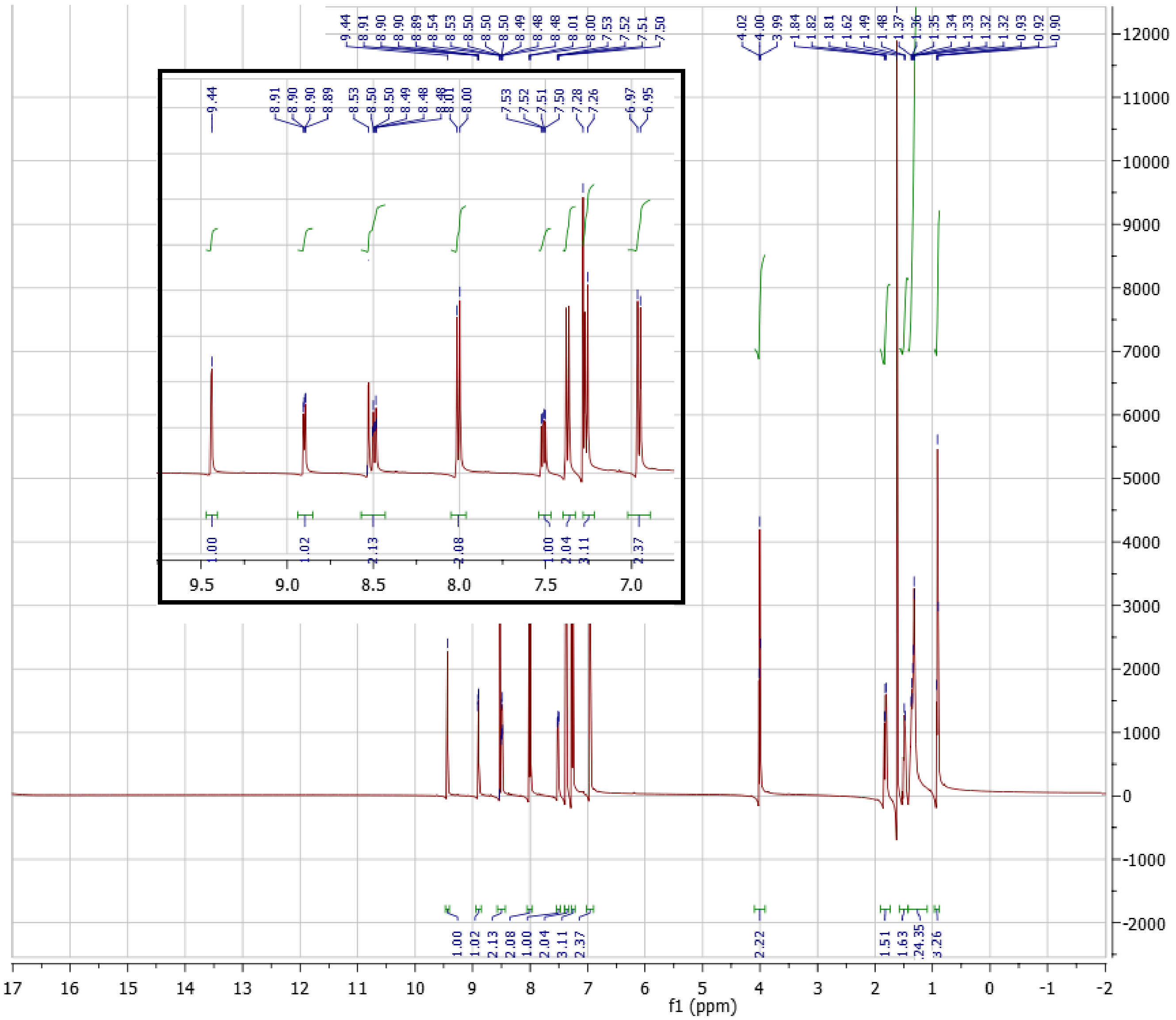

Figure S2. ^{1}HNMR of Pyridin−2−yl 4−[4−(hexadecyloxy)phenyliminomethyl]benzoate (**I C16**).
